# Green synthesis of ZnO/Fe_3_O_4_ nanocomposites from *Citrus reticulata* peel: antibacterial activity against MDR *Acinetobacter baumannii*, CRISPR-Cas gene modulation, and anticancer potential

**DOI:** 10.1039/d6ra02362b

**Published:** 2026-07-02

**Authors:** Noor Majed Abdallah, Mohammed Rasheed Mohammed, Halah Al Haideri, Amirah S. Alahmari, Khairiah M. Alwutayd, Rowa K. Zarah, Hamida Hamdi, Luluah M. Al Masoudi, Noha T. Al Thagafi, Ashwaq T. Althobaiti, Mohsen A. Khormi, Abdulkarim S. Binshaya, Adil Abalkhail, Mohamed K. Y. Soliman

**Affiliations:** a Department of Biology, College of Science for Women, University of Baghdad Baghdad 10071 Iraq; b Al-Khwarizmi College of Engineering, University of Baghdad Iraq; c Department of Biology, College of Science, Princess Nourah bint Abdulrahman University P.O. Box 84428 Riyadh 11671 Saudi Arabia; d Department of Biology, College of Science, Taif University P.O. Box 11099 Taif 21944 Saudi Arabia; e Zoology Department, Faculty of Science, Cairo University Giza 12613 Egypt; f Department of Biology, College of Science, Jazan University P.O. Box. 114 Jazan 45142 Kingdom of Saudi Arabia; g Department of Medical Laboratory, College of Applied Medical Sciences, Prince Sattam Bin Abdulaziz University 11942 Alkharj Saudi Arabia; h Department of Public Health, College of Applied Medical Sciences, Qassim University P.O. Box 6666 51452 Buraydah Saudi Arabia; i Botany and Microbiology Department, Faculty of Science, Al-Azhar University Nasr City Cairo 11884 Egypt Mohamed.k.yousef@azhar.edu.eg

## Abstract

The emergence of multidrug-resistant (MDR) *Acinetobacter baumannii* necessitates the development of alternative antimicrobial strategies. In this study, ZnO/Fe_3_O_4_ nanocomposites (NCs) were green-synthesized using *Citrus reticulata* peel extract and evaluated for antibacterial, CRISPR-Cas gene modulation, anticancer, and antioxidant activities. Phytochemical profiling by GC-MS and HPLC confirmed a terpene- and polyphenol-rich composition supporting nanoparticle formation and stabilization. The synthesized NCs were characterized by UV-vis, FTIR, and XRD analyses, supporting the formation of crystalline ZnO and Fe_3_O_4_ phases, while TEM revealed nanoscale morphology (55.64 ± 24.2 nm) with hydrodynamic size of ∼181.3 nm and a zeta potential of +2.64 mV. Fifteen clinical *A. baumannii* isolates were identified, among which nine exhibited multidrug-resistant (MDR) profiles. CRISPR-associated genes were screened in the MDR isolates, and four isolates harboring the target genes were selected for further molecular analyses. The ZnO/Fe_3_O_4_ NCs exhibited antibacterial activity with inhibition zones ranging from 17 to 24 mm and MIC values of 250–500 µg mL^−1^. TEM analysis of treated bacteria demonstrated severe structural damage, including membrane disruption and cytoplasmic leakage. Furthermore, sub-MIC exposure resulted in downregulation of CRISPR-associated genes (Cas1: 0.61–0.98; Csy1: 0.54–0.95; Csy3: 0.70–0.83). Cytotoxicity assays revealed selective antiproliferative effects against Caco-2 colorectal cancer cells (IC_50_ = 60.7 µg mL^−1^) compared to normal Vero cells (IC_50_ = 254.66 µg mL^−1^), accompanied by increased apoptosis (15.8%) and G2/M cell cycle arrest (39.7%). Additionally, the NCs exhibited concentration-dependent antioxidant activity, reaching up to 69.44% (DPPH), 76.12% (ABTS), 62.24% (H_2_O_2_ scavenging), 56.13% (metal chelation), and 63.52% (reducing power). Overall, these findings demonstrate that green-synthesized ZnO/Fe_3_O_4_ NCs are multifunctional nanomaterials with promising antibacterial, anticancer, and antioxidant properties.

## Abstract 2

Green-synthesized ZnO/Fe_3_O_4_ nanocomposites derived from *Citrus reticulata* peel exhibit antibacterial activity against MDR *Acinetobacter baumannii*, modulate CRISPR-Cas genes, induce anticancer effects, and possess antioxidant properties.

## Graphical abstract

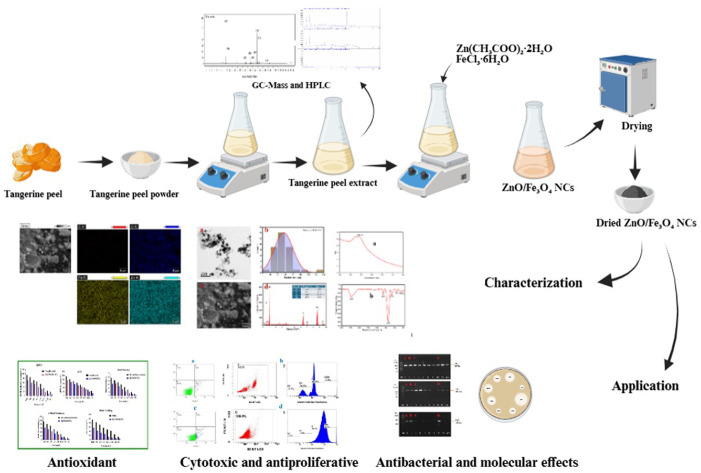

## 1 Introduction

The rapid dissemination of multidrug-resistant (MDR) bacterial strains across the globe poses a substantial risk to human health, significantly limiting the effectiveness of conventional antibiotic therapies.^1,2^ Concern has been directed toward *Acinetobacter baumannii*, given its high propensity to develop multidrug resistance and endure in hospital environments, contributing to severe infections such as UTIs, bacteremia, and ventilator-associated pneumonia. Recent global surveillance data indicate an alarming increase in carbapenem-resistant *A. baumannii* isolates, especially in intensive care settings, where therapeutic options are extremely limited.^3^ In addition to classical resistance mechanisms, such as efflux pumps, β-lactamase production, and alterations in membrane permeability, adaptive immune systems, including CRISPR-Cas, have been implicated in bacterial genomic plasticity and resistance regulation. Recent studies suggest that CRISPR-associated systems in *A. baumannii* may influence horizontal gene transfer, virulence, and stress adaptation under antimicrobial pressure.^4^ Therefore, novel strategies that not only inhibit bacterial growth but also modulate resistance-associated genetic systems are urgently needed. Nanotechnology-based antimicrobial approaches have shown promising alternatives due to their multi-target mechanisms of action and reduced likelihood of resistance development.^5,6^ Globally, colorectal cancer continues to be one of the primary causes of cancer-related death.^7^ Treatment resistance and systemic toxicity continue to be significant clinical difficulties in spite of advancements in targeted therapy and chemotherapy. Nanoparticle-based therapeutic systems offer innovative solutions by selectively targeting cancer cells through ROS-mediated apoptosis, mitochondrial damage, and cell cycle arrest mechanisms.^8^

Metal oxide nanoparticles, particularly ZnO and Fe_3_O_4_, have attracted significant interest in biomedical research because of their distinct physicochemical aspects, such as their large surface area, redox activity, and tunable surface chemistry. ZnO nanoparticles are well known antimicrobial and antioxidants, membrane disruption, and mitochondrial dysfunction.^6^ Similarly, Fe_3_O_4_ nanoparticles exhibit magnetic responsiveness, biocompatibility, and may influence cellular uptake, making them valuable for targeted therapeutic applications.^9^ Current developments in nanotechnology have concentrated on creating bimetallic or hybrid nanocomposites, such as ZnO/Fe_3_O_4_ systems, which combine the functional advantages of both components. These nanocomposites have demonstrated enhanced antibacterial and anticancer efficacy compared to single-metal nanoparticles due to synergistic interactions between metal oxides.^10–12^ Recent investigations have demonstrated that ZnO-based nanostructures induce selective cytotoxicity in colorectal cancer cells while exhibiting comparatively lower toxicity toward normal cells.^13^

Importantly, sustainable fabrication approaches utilizing botanical extracts have gained prominence as eco-friendly, cost-effective, and viable substitutes for traditional techniques in chemical synthesis. Plant-derived phytochemicals, including phenolic acids, flavonoids, and terpenoids, act as reducing, stabilizing, and capping agents, improving nanoparticle stability and biological performance.^14–16^ Recent studies highlight that plant-mediated ZnO-based nanocomposites exhibit improved bioactivity and reduced toxicity profiles compared to chemically synthesized counterparts.^17^ The combination of Fe_3_O_4_ with ZnO in a nanocomposite system may enhance intracellular uptake and oxidative stress induction due to the synergistic physicochemical properties of both components, thereby improving therapeutic efficacy. Moreover, beyond their antimicrobial and anticancer activities, ZnO/Fe_3_O_4_ NCs have demonstrated significant antioxidant potential through radical scavenging, hydrogen peroxide neutralization, and metal ion chelation mechanisms, which are largely influenced by surface-bound phytochemicals in green-synthesized systems.^18^

*Citrus reticulata* peel (tangerine peel) represents an abundant agro-industrial byproduct enriched with diverse bioactive phytochemicals that possess significant pharmacological and technological importance. Comprehensive phytochemical investigations have confirmed the richness of citrus peels in flavonoids such as hesperidin, naringin, quercetin, and epicatechin, in addition to various phenolic acids, including gallic, chlorogenic, gentisic, and *p*-hydroxybenzoic acids.^19^ These compounds exhibit strong antioxidant potential due to their electron-donating hydroxyl groups and redox-active structures. Moreover, citrus peel essential oils are dominated by monoterpenes, particularly d-limonene and γ-terpinene, which contribute to antimicrobial and membrane–disruptive activities against pathogenic microorganisms.^20^ Recent research has demonstrated the crucial function of phenolic hydroxyl and carboxyl functional groups in reducing metal ions and stabilizing biosynthesized nanoparticles during green fabrication processes.^21^ The presence of these phytochemicals facilitates nucleation, surface functionalization, and enhanced colloidal stability of metal and metal oxide nanostructures. Furthermore, citrus peel-derived compounds have demonstrated synergistic antioxidant, antimicrobial, and anticancer effects when incorporated into nanostructured systems.^22^ Owing to its rich biochemical profile, environmental sustainability, and low cost, *Citrus reticulata* peel has emerged as a promising natural resource for eco-friendly synthesis of multifunctional nanomaterials with improved biological performance.^23^

Given these multifunctional properties, exploring plant-mediated ZnO/Fe_3_O_4_ NCs as dual antibacterial and anticancer agents represents a promising research direction. However, limited studies have investigated their impact on CRISPR-associated gene expression in MDR bacteria while simultaneously evaluating their cytotoxic and antioxidant performance. Therefore, the current study aimed to synthesize ZnO/Fe_3_O_4_ NCs using *Citrus reticulata* peel extract and to comprehensively evaluate their physicochemical characteristics, antibacterial activity, modulation of CRISPR-associated genes, anticancer effects against Caco-2 cells, and antioxidant potential. The novelty of this study lies in the integrated evaluation of CRISPR-Cas gene modulation alongside antibacterial activity against MDR *Acinetobacter baumannii*, combined with anticancer and antioxidant assessments within a single green-synthesized ZnO/Fe_3_O_4_ NCs system.

## 2 Materials and methods

### 2.1. Materials

All of the analytical-grade chemicals and reagents used in this investigation were acquired from Sigma-Aldrich (St. Louis, MO, USA), including the precursor materials for nanocomposite synthesis. Cell culture media were purchased from HiMedia Laboratories (Giza, Egypt). MTT (3-(4,5-dimethyl-2-thiazolyl)-2,5-diphenyl-2*H*-tetrazolium bromide), paclitaxel (Taxol), and dimethyl sulfoxide (DMSO) were obtained from Sigma Chemical Co. (St. Louis, MO, USA). Fetal bovine serum (FBS), phosphate-buffered saline (PBS), trypsin-EDTA, Dulbecco's Modified Eagle's Medium (DMEM) (Thermo Fisher Scientific, USA). Deionized distilled water was used in all biosynthesis and experimental procedures.

### 2.2. Collection of plant materials

Fresh tangerine fruit was purchased from nearby local markets and thoroughly washed with sterile distilled water. The peels were then removed, chopped into smaller pieces, and cleaned to eliminate any residual dirt or debris. To remove moisture, the chopped peels were dried for four to five days at a temperature of 40 °C for six to seven hours daily. Once dried. The peels were finely powdered using a mixer grinder and stored for future use.

### 2.3. Preparation of tangerine peel extract

The tangerine peel extract was prepared using the developed tool from Lourenço *et al.* (2021).^24^ Approximately 10 g of dried peel Powder was combined with 250 mL of deionized distilled water (DDW) and heated to 70–80 °C for around 45 minutes on a heating mantle. After cooling, the solution was purified *via* vacuum filtration. Subsequently, the filtrate was centrifuged at 16 000 rpm for 15 minutes at room temperature, and the remaining supernatant was stored at low temperatures for later use.

### 2.4. Green biosynthesis of ZnO/Fe_3_O_4_ NCs

Tangerine peel extract was employed as a stabilizing and lowering agent in the green synthesis of ZnO/Fe_3_O_4_ NCs. Briefly, 60 mL of the prepared extract was heated to 60 °C while being continuously stirred by a magnet at 250 rpm in a thermostatically controlled reaction vessel. Zinc acetate dihydrate (Zn(CH_3_COO)_2_·2H_2_O) and ferric chloride hexahydrate (FeCl_3_·6H_2_O) were used to create aqueous precursor solutions. The metal salt solutions were prepared at a final concentration of 25 mM. An 8 mL aliquot of the mixed metal precursor solution was added dropwise to the heated plant extract under constant stirring. The reaction mixture was maintained under stirring for an additional 2 h to facilitate the reduction and nucleation of ZnO/Fe_3_O_4_ NCs. The resulting suspension was then cooled and stored at 4 °C for 48 h to allow complete formation and stabilization of the nanocomposite particles. The synthesized ZnO/Fe_3_O_4_ NCs were collected by centrifugation at 16 000 rpm for 15 min at 4 °C. The obtained pellet was washed repeatedly with deionized water to remove unreacted residues and then dried in a hot-air oven at 200 °C until constant weight for further characterization and biological evaluation.

### 2.5. Gas chromatography-mass spectrometry (GC/MS) analysis

A Thermo Scientific Trace GC1310-ISQ mass spectrometer (Austin, TX, USA) fitted with a TG-5MS capillary column (30 m × 0.25 mm × 0.25 µm film thickness) was used to undertake chemical profiling of the samples. The oven program initiated at 50 °C, ramping up to 230 °C at 5 °C per minute, then increasing at a rate of 30 °C per minute to an end temperature of 290 °C, maintained for two minutes. Helium was used as the carrier gas at a flow rate of 1 mL min^−1^; the injector and MS transfer line temperatures were set at 250 °C and 260 °C, respectively. The volume of one microliter of each diluted sample was injected using an AS1300 autosampler in split mode. In full-scan mode, mass spectrometric detection was carried out using a scanning mass range of *m*/*z* 40–1000 under electron ionization (EI) at 70 eV. Compound identification was done by comparing retention durations and mass spectra to the WILEY 09 and NIST 11 reference libraries.^25^

### 2.6. High-performance liquid chromatography (HPLC) analysis

High-performance liquid chromatography was performed following the method of Kim *et al.*^26^ using an Agilent 1260 Infinity II LC system equipped with a diode-array detector and autosampler. An Eclipse XDB-C18 analytical column (150 × 4.6 mm, 5 µm particle size), coupled with a C18 guard column, was employed for separation. The elution system consisted of acetonitrile (solvent A) and 2% acetic acid solution (solvent B). Gradient elution progressed from 100% B to 85% B over 30 minutes, then to 50% B during the subsequent 20 minutes, followed by a shift to 0% B in 5 minutes and reconditioning back to 100% B within 5 minutes. The chromatographic run lasted 60 minutes at a constant flow rate of 0.8 mL min^−1^. A 50 µL sample volume was injected. Monitoring was carried out at 280 and 320 nm for benzoic and cinnamic acid derivatives, while flavonoids were detected at 360 nm. All samples underwent filtration through 0.45 µm membrane filters before analysis. Peak assignment was achieved through comparison with authentic standards based on retention behavior and UV spectral characteristics.

### 2.7. Characterization of ZnO/Fe_3_O_4_ NCs

Spectrophotometry (JASCO V 630, Hachioji, Japan) was employed to detect absorption peaks corresponding to the surface plasmonic resonance of the synthesized ZnO/Fe_3_O_4_ NCs across the UV-visible range (200–800 nm). Transmission electron microscopy (JEOL 2100, Akishima, Japan) was utilized to examine the size and morphology of the nanoparticles. Fourier Transform Infrared (FT-IR) spectroscopy (JASCO, FT/IR-6100) was performed to assess functional groups, with generated ZnO/Fe_3_O_4_ NCs compressed into discs combined with potassium bromide (KBr) for analysis in the 400–4000 cm^−1^ range. Additionally, the crystalline nature of the nanoparticles was determined through X-ray diffraction (XRD) using a Philips XRD system, analyzing the crystalline form over two hours between 10° and 80°. Scanning electron microscopy (SEM, ZEiSS, EVO-MA 10, Oberkochen, Germany) further assessed size and shape, while energy-dispersive X-ray spectroscopy (EDX BRUKER, Nano GmbH, D-12489, M410, Germany) was employed to evaluate the elemental composition, purity, and dispersion of the nanoparticles. Dynamic Light Scattering (DLS) using the Zetasizer Nano Series (Nano ZS, Malvern Instruments, UK) was used to measure the particle size distribution. A NICOMP™ 380 ZLS analyzer operating with phase-analysis light scattering at a wavelength of 1.54 nm and a count rate of 286 kHz at 30 °C was used to assess surface charges utilizing zeta potential analysis.^27,28^

### 2.8. Identification of microorganisms

Between September and December 2025, fifteen samples were collected from patients at Baghdad Medical City Hospital in Baghdad, Iraq, diagnosed with urinary tract infections. The *Acinetobacter baumannii* species were identified using biochemical tests and selective agar media. The suspected colonies were morphologically confirmed with the Vitek 2 compact system (BioMerieux, France).

### 2.9. Antibiotic susceptibility testing

Antimicrobial susceptibility testing of the confirmed *Acinetobacter baumannii* isolates was carried out using the Antibiotic Susceptibility Test Card (AST-N222 Supplement) with the VITEK 2 Compact system (bioMérieux, France). The following antibiotics were tested: Trimethoprim/Sulfamethoxazole, Ciprofloxacin (CIP-10), Levofloxacin (LEV-5), Chloramphenicol + Ofloxacin (C + O-10), Cefotaxime (CTX-10), Ceftazidime (CAZ-30), Amikacin (AK-10), Gentamicin (GEN-10), Tobramycin (TOB-10), Piperacillin + Tazobactam (P + L-100), Imipenem (IPM-10), and Doxycycline (DO-10). Four isolates were a Multidrug-resistant (MDR strains of *Acinetobacter baumannii* selected to investigate the ZnO/Fe_3_O_4_ NCs activity.

### 2.10. Agar well diffusion assay

To investigate the antibacterial potential of ZnO/Fe_3_O_4_ NCs against four MDR *Acinetobacter baumannii* isolates, the agar well diffusion method was employed. A standardized bacterial inoculum (0.5 McFarland) was prepared, and 100 µL was evenly spread over Mueller–Hinton agar plates using sterile cotton swabs. Sterile cork borers were used to create wells of 6 mm diameter in the agar medium. Subsequently, 100 µL of ZnO/Fe_3_O_4_ NCs solution (2000 µg mL^−1^) was added to each well. Colistin was used as a positive control, while *Citrus reticulata* peel extract alone was used as a control under the same conditions. Plates were incubated at 37 °C for 18–24 h. After incubation, the diameters of inhibition zones were measured in millimeters to evaluate antibacterial activity. All experiments were performed in triplicate, and mean values were calculated.

### 2.11. Minimum inhibitory concentration of ZnO/Fe_3_O_4_ NCs

The broth microdilution technique in culture broth was used to assess the synthesized ZnO/Fe_3_O_4_ NCs' minimum inhibitory concentration (MIC) against isolated *A. baumannii*. Serial dilutions of 1000, 500, 250, 125, 62.5, and 31.25 µg mL^−1^ were prepared from a base solution of 1000 µg mL^−1^ of NPs. The first row of wells in a microtiter plate was filled with 100 µL of the diluted solution, followed by 100 µL of Müller Hinton broth (MHB) and the ZnO/Fe_3_O_4_ NCs solution in each well. The mixture was incubated for 18 to 24 hours. Wells 1 through 10 were then established using a two-fold dilution method, with one acting as a positive control and another as a negative control (MHB alone). Each well, except for the negative control, received an inoculum of bacterial culture with a McFarland standard adjustment (1.5 × 10^8^ CFU mL^−1^). Following that, 30 µL of resazurin solution (0.02 percent wt per v) (HiMedia) was added to each well of the plate, and it was incubated once again for 6 hours in order to identify bacterial growth. A change in hue from blue to red signified the presence of bacterial growth. The growth control wells' color changed to red when the strains were grown correctly, while a sterile control well's color remained unchanged when contamination was absent. The lowest concentration of ZnO/Fe_3_O_4_ NCs with no bacterial growth was identified as the MIC.^29^

### 2.12. RT-qPCR protocol

RNA was extracted from samples using the TRIzol™ Reagent protocol, which included the following steps:

(1) Sample lysis: suspension-grown cells were centrifuged at 13 000 rpm for two minutes. After discarding the supernatant, the pellet was mixed with 0.5 mL of TRIzol™ Reagent and pipetted repeatedly to homogenize it.

(2) Phase separation: chloroform (0.2 mL) was added to each tube, sealed tightly, and then centrifuged at 12 000 rpm for ten minutes after a two to three-minute incubation. This procedure yielded three phases: a lower organic phase, an interphase, and a clear upper aqueous phase, from which the RNA-containing aqueous phase was transferred to a new tube.

(3) RNA precipitation: isopropanol (0.5 mL) was introduced into the aqueous phase, incubated for ten minutes, and then centrifuged for an additional ten minutes at 12 000 rpm. The supernatant was discarded, leaving a gel-like white RNA pellet at the bottom of the tube.

### 2.13. Detection of CRISPR genes (Csy1, Csy3, and Cas1)

Genomic DNA was extracted from bacterial cultures utilizing the ABIO pure extraction method. Primers supplied by Macrogen Company were lyophilized and subsequently dissolved in nuclease-free water to reach a concentration of 100 pmol µL^−1^. A working solution of 10 pmol µL^−1^ was prepared by mixing 10 µL of the stock solution with 90 µL of nuclease-free water and stored at −20 °C. The primer sequences used in this study are listed in Table S2. Following particular standards for the extracted DNA, agarose gel electrophoresis was used to confirm the existence of the amplified product after PCR amplification. PCR products were loaded, and the gel was run at 100 V for 60 minutes to visualize ethidium bromide-stained bands using gel imaging equipment.^30,31^

### 2.14. Cell culture and MTT assay

Vero (African green monkey kidney) and Caco-2 (human colorectal carcinoma) cell lines were sourced from ATCC (LGC Promochem, UK) one day before conducting the assay. Cells were detached using trypsin/EDTA, counted, and seeded in 96-well plates at 8 × 10^3^ cells per well in 200 µL DMEM supplemented with 10% FBS, penicillin G (10 000 IU), streptomycin (10 mg), and amphotericin B (25 µg). Cultures were maintained at 37 °C with 5% CO_2_ for 24 h to ensure proper adhesion. Cancer cells were exposed to graded concentrations (15.62 to 1000 µg mL^−1^) of the tested formulations, whereas untreated control cells received 0.1% DMSO. After 72 h, cell viability was determined using the MTT assay (Vybrant®, Thermo Fisher). Fresh medium containing 20 µL MTT reagent (1 mg mL^−1^) was added to each well and incubated for 4 h. Subsequently, the medium was discarded and 100 µL SDS-HCl solution was added to solubilize the formazan product. Optical density was recorded at 570 nm using a Bio-Tek ELx800 microplate reader.^32^

### 2.15. Assessment of apoptosis by annexin V/PI staining in cultured cells

Apoptosis was evaluated using the Alexa Fluor® 488 Annexin V/Dead Cell Apoptosis Kit (Invitrogen, UK) according to the manufacturer's instructions.^33^ Briefly, cells were washed twice with phosphate-buffered saline (PBS) and centrifuged at 400×*g* for 5 min. The cell pellet was gently resuspended in 1X annexin-binding buffer, and 5 µL of Alexa Fluor® 488 Annexin V and 1 µL of propidium iodide (PI, 100 µg mL^−1^ working solution) were added to each 100 µL of cell suspension. Samples were incubated for 15 min at room temperature in the dark. Subsequently, 400 µL of 1X annexin-binding buffer was added, and samples were kept on ice until analysis. Stained cells were analyzed by flow cytometry using 488 nm excitation, and fluorescence emissions were collected at 530 nm (Annexin V-FITC) and 575 nm (PI). Data acquisition and analysis were performed using Beckman Coulter Navios EX software.

### 2.16. Assessment of cell cycle using DNA staining by flow cytometry

After 48 hours of treatment, collected cells were scraped, fixed with cold methanol, and stained with Vybrant DyeCycle Violet stain (Invitrogen, cat no. V35003), then analyzed *via* flow cytometry to evaluate DNA content across the cell cycle. A concentration of 5 µM of Vybrant® DyeCycle™ Violet stain was prepared by mixing 1 µL with 1 mL of cell suspension at a concentration of 1 × 10^6^ cells per mL. The stained cells were kept out of the light until they were acquired after being incubated for 30 minutes at 37 °C. Beckman Coulter Navios EX software (version: SM: BE14548) was used to analyze the data, with particular attention paid to the distribution of apoptotic sub-G1 cells, G0/G1 phase, S phase, and G2M phase.

### 2.17. Antioxidant activity assays

#### 2.17.1. DPPH free radical scavenging assay

The antioxidant capacity of the ZnO/Fe_3_O_4_ NCs was assessed using the DPPH radical scavenging method with minor adjustments. In brief, 1.0 mL of a freshly prepared ethanolic DPPH solution (0.2 mg mL^−1^; 0.135 mM) was placed into test tubes. There were different concentrations of the sample (7.81–1000 µg mL^−1^), and combined with the DPPH solution. The mixes were left to react for 30 minutes at room temperature in the dark after being gently stirred for 1 minute. After incubation, a microplate reader (Epoch 2 Microplate Spectrophotometer, BioTek, USA) was used to measure absorbance decrease at 517 nm, using ethanol as the blank. Ascorbic acid was used as a reference antioxidant under identical experimental conditions.

#### 2.17.2. ABTS radical cation decolorization assay

The ABTS radical scavenging capacity of the ZnO/Fe_3_O_4_ NCs was determined using a modified ABTS˙^+^ discoloration assay. After mixing ABTS (7 mM) and potassium persulfate (2.5 mM) in equal volumes, the mixture was let to remain at room temperature in the dark for 16 hours to produce the radical cation. The resulting ABTS working solution was then combined with different concentrations of the extract (7.81–1000 µg mL^−1^) and incubated briefly under dark conditions. The reference antioxidant was ascorbic acid. A microplate reader was used to measure absorbance at 734 nm.^29^

#### 2.17.3. Metal chelating assay method

The ability of the ZnO/Fe_3_O_4_ NCs to chelate ferrous ions was assessed using the ferrozine-based method with minor modifications. In brief, 0.5 mL of FeCl_2_ solution (0.2 mM) was mixed with different concentrations of the tested sample (7.81–1000 µg mL^−1^). In order to allow the ferrozine–Fe^2+^ complex to form, the reaction was started by adding 0.2 mL of ferrozine solution (5 mM) and then allowed to sit at room temperature for 10 to 15 minutes. EDTA was employed as a reference chelating agent. A microplate reader was used to measure absorbance at 562 nm.

#### 2.17.4. Evaluation of hydroxyl radical scavenging potential

The capacity of the ZnO/Fe_3_O_4_ NCs was evaluated with a modified deoxyribose assay. The reaction mixture was prepared in phosphate buffer (20 mM, pH 7.4) containing FeCl_3_ (1 mM), EDTA (1 mM), deoxyribose (2.8 mM), and hydrogen peroxide (20 mM). Radical generation was initiated by adding ascorbic acid (1 mM). The extract, tested at concentrations between 7.81 and 1000 µg mL^−1^, was incubated with the reaction system at 37 °C for 1 h. After incubation, thiobarbituric acid (1%) and trichloroacetic acid (2.8%) were introduced, and the mixtures were heated at 100 °C for 20 min. A microplate reader was used to measure the cooled samples' absorbance at 532 nm, with Butylated hydroxytoluene (BHT) as a standard antioxidant.

#### 2.17.5. Ferric reducing power assay

The reducing ability of the ZnO/Fe_3_O_4_ NCs was determined using a ferricyanide-based assay. Potassium ferricyanide (1%) and phosphate buffer (0.2 M, pH 6.6) were combined with extract solutions (7.81–1000 µg mL^−1^) and incubated for 20 minutes at 50 °C. After stopping the reaction with 10% trichloroacetic acid, the mixture was centrifuged for 10 minutes at 1750 rpm. The supernatant was diluted with deionized water (1 : 1, v/v), then reacted with ferric chloride (0.1%). After incubation at 35 °C for 10 min, absorbance was recorded at 700 nm. Reducing power was estimated from absorbance values, using butylated hydroxytoluene (BHT) as the reference antioxidant.

### 2.18. Statistical analysis

Statistical analysis was performed using GraphPad Prism 8 software. Data are presented as mean ± standard deviation (SD) from three independent biological replicates. Where applicable, each experiment was conducted with technical replicates, and the average values were used for statistical analysis. Differences between groups were analyzed using two-way analysis of variance (ANOVA) followed by Tukey's post hoc test. A *p*-value of less than 0.05 was considered statistically significant.

## 3 Results and discussion

### 3.1. GC/MS and HPLC analysis of polyphenolic compounds in tangerine peel extract

GC-MS analysis of the tangerine peel extract (Fig. S1 and Table S3) revealed a phytochemical profile predominantly composed of monoterpenes and sesquiterpenes, which are typical constituents of citrus peels. The extract was mainly dominated by d-limonene (23.66%), followed by germacrene D (20.57%) and β-cadinene (14.81%), confirming the terpene-rich nature of the extract. The high abundance of d-limonene is consistent with recent reports on citrus peel extracts and is considered a key contributor to their antioxidant and antimicrobial activities.^34^ Other monoterpenes, including γ-terpinene (6.82%), *p*-cymene (3.88%), and α-terpinene (0.94%), were also detected. These compounds are known for their free radical scavenging ability and antimicrobial potential through membrane disruption and oxidative stress induction in microbial cells.^20^ The presence of thymol (2.74%), a phenolic monoterpene, further enhances the biological relevance of the extract, as thymol has been widely reported to exhibit strong antibacterial and antibiofilm activities.^35^ The sesquiterpene fraction, represented by copaene, humulene, caryophyllene, bicyclogermacrene, and cadinene derivatives, plays an important role in the biological functionality of the extract. Sesquiterpenes have been reported to exert antimicrobial and anti-inflammatory effects, often acting synergistically with monoterpenes to enhance overall bioactivity.^36^ Additionally, oxygenated compounds such as *p*-vinylguaiacol and shyobunol may contribute to antioxidant capacity and functional properties of the extract.

HPLC study of the aqueous tangerine (*Citrus reticulata*) peel extract revealed a diverse and abundant phenolic profile (Fig. S2 and S3), confirming its suitability as a reducer for green nanoparticle synthesis. Gallic acid was identified as the predominant phenolic compound, with a concentration of 2126.80 µg g^−1^, indicating its major contribution to the antioxidant and reducing capacity of the extract. Recent studies have reported gallic acid as a key phenolic component in citrus peels, highlighting its strong electron-donating and metal-chelating properties, which play a crucial role in nanoparticle nucleation and stabilization.^21,37^ Other phenolic acids, including gentisic acid (678.21 µg g^−1^), *p*-hydroxybenzoic acid (310.32 µg g^−1^), chlorogenic acid (186.31 µg g^−1^), and vanillic acid (55.74 µg g^−1^), were detected in considerable amounts. These compounds are known to act synergistically with gallic acid, enhancing the redox potential of plant extracts and facilitating the formation of stable metal and metal oxide nanoparticles.^38^ The presence of these acids also contributes to the biological activity of the synthesized nanoparticles due to their inherent antimicrobial and antioxidant properties. Flavonoid analysis revealed high levels of epicatechin (342.70 µg g^−1^) and hesperidin (199.33 µg g^−1^), along with moderate concentrations of catechin (42.61 µg g^−1^), apigenin-7-glucoside (68.16 µg g^−1^), quercetin (24.47 µg g^−1^), and rosmarinic acid (16.10 µg g^−1^). Hesperidin, a citrus-specific flavanone glycoside, has been widely reported in recent literature for its anticancer, antioxidant, and antimicrobial activities, particularly when incorporated into nanostructured systems.^39^ The absence of certain phenolics such as caffeic acid, ferulic acid, rutin, apigenin, and kaempferol may be attributed to differences in citrus cultivar, geographical origin, and extraction conditions, as reported in recent citrus phytochemical studies.^19^ The phenolic-rich composition of the tangerine peel extract provides a strong biochemical explanation for its effectiveness in the fabrication of NCs. Phenolic hydroxyl and carboxyl groups can bind metal ions, promote reduction, and form an organic capping layer on the nanoparticle surface, enhancing stability and biological performance. This mechanism is consistent with recent reports on botanical extract-mediated synthesis of metal oxide nanocomposite materials.^40^ Moreover, the identified phenolic acids and flavonoids have been shown to potentiate antimicrobial and anticancer activities through membrane disruption, induction of oxidative stress, and modulation of apoptotic pathways. Recent studies demonstrated that phenolic-functionalized nanoparticles exhibit enhanced activity against multidrug-resistant bacteria and improved cytotoxic selectivity toward cancer cells compared with bare nanoparticles.^15,41^ Therefore, the observed biological activities of the synthesized ZnO/Fe_3_O_4_ NCs can be partially attributed to the surface-bound phytochemicals derived from the tangerine peel extract.

### 3.2. Characterization of the biosynthesized ZnO/Fe_3_O_4_ NCs

#### 3.2.1. UV-vis, FTIR and XRD characterization studies

**Fig. 1:**
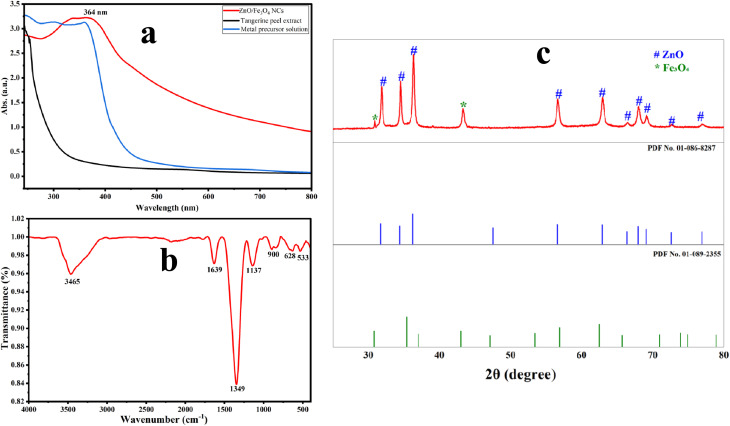
UV-vis spectra of ZnO/Fe_3_O_4_ NCs, tangerine peel extract, and metal precursor solution (a); FTIR spectrum (b) and XRD analysis (c) of ZnO/Fe_3_O_4_ NCs by tangerine peel extract.

The capacity of tangerine peel extract to promote the biosynthesis of ZnO/Fe_3_O_4_ NCs was investigated by UV-visible spectrophotometry. The UV-vis spectrum of the synthesized ZnO/Fe_3_O_4_ NCs showed a distinct absorption band at 364 nm (Fig. 1a) indicating successful formation of the nanocomposite and the development of characteristic optical features associated with nanoscale metal oxide systems. The pronounced intensity of the deep dark brown color of the tangerine peel extract aligns with its effectiveness in biosynthesizing bimetallic ZnO/Fe_3_O_4_ NCs. Comparative UV-vis spectra of the *Citrus reticulata* peel extract and metal precursor solution were additionally analyzed under identical experimental conditions. The synthesized ZnO/Fe_3_O_4_ NCs exhibited a broader and more pronounced absorption feature around 364 nm compared with the extract and precursor solution, supporting the formation of the nanocomposite system. Previous investigations have shown that the formation of iron nanoparticles can be identified in UV-vis spectra by a characteristic absorption band around 287 nm, along with additional signals appearing within the 280–300 nm region. These absorption features are considered indicative of nanoscale iron formation and are commonly associated with electronic transitions occurring in iron-based nanostructures.^42^ In the case of ZnO nanoparticles, a pronounced absorption peak centered near 360 nm is typically observed, suggesting nanoparticle formation. The emergence of a band within the 360–380 nm range is typically attributed to intrinsic optical properties related to Zn-based nanomaterials, confirming particle formation and growth. Supporting this trend, Mirzaei *et al.*^43^ described the biosynthesis of ZnSe nanoparticles, where a visible color change in the reaction mixture initially suggested nanoparticle formation. Their UV-vis analysis revealed two distinct absorption peaks at approximately 250 nm and 360 nm, confirming the successful production of ZnSe nanoparticles using seaweed extract as a reducing agent. The observed absorption band is consistent with previous reports on ZnO nanoparticles, where characteristic UV-vis peaks in the 350–380 nm range confirm successful nanoparticle formation and optical properties.^44^

Fourier Transform Infrared spectroscopy was employed to identify the functional groups and chemical interactions present in the synthesized ZnO/Fe_3_O_4_ NCs. The spectrum, presented in Fig. 1b, was recorded over a wavenumber range of 400–4000 cm^−1^. The FTIR profile of the bimetallic nanocomposite displays several characteristic absorption bands located at approximately 533, 628, 900, 1137, 1349, 1639, and 3465 cm^−1^, reflecting the presence of both organic residues from the plant extract and metal–oxygen vibrations. The broad and intense band centered around 3465 cm^−1^ is attributed to the stretching vibration of hydroxyl (–OH) groups, which may arise from adsorbed water molecules or phenolic constituents derived from the plant extract. The absorption peak observed at 1639 cm^−1^ can be assigned to C=C stretching vibrations, indicating the presence of unsaturated organic compounds. Meanwhile, the band near 1349 cm^−1^ corresponds to C–H bending vibrations associated with aliphatic groups. The signal detected at 1137 cm^−1^ is likely related to C–O stretching, possibly originating from amide or other oxygen-containing functional groups present in the biomolecules used during synthesis.^45^ Importantly, strong absorption bands appearing at 628 and 533 cm^−1^ confirm the formation of Zn–O bonds, which are characteristic of ZnO nanostructures. In general, vibrational modes observed within the 500–800 cm^−1^ region are indicative of metal–oxygen stretching, further supporting the successful coordination between zinc ions and oxygen during nanoparticle formation.^46^ These findings collectively verify the successful synthesis of ZnO/Fe_3_O_4_ NCs and highlight the role of bioactive functional groups in stabilizing the nanostructured system.

The crystal structure and phase composition of the synthesized ZnO/Fe_3_O_4_ NCs were investigated using XRD analysis with CuKα radiation (*λ* = 1.5406 Å). The obtained diffraction pattern (Fig. 1c) exhibits well-defined peaks corresponding to both ZnO and Fe_3_O_4_ phases, indicating the formation of a biphasic nanocomposite system. The prominent diffraction peaks observed at 2*θ* values of 31.88°, 34.53°, 36.36°, 56.66°, 62.93°, 66.57°, 67.78°, 69.13°, 72.73°, and 76.97° were indexed to the (100), (002), (101), (110), (103), (200), (112), (201), (004), and (202) crystallographic planes of hexagonal wurtzite ZnO, respectively. These reflections are in good agreement with the standard reference pattern for ZnO (PDF No. 01-086-8287), confirming the preservation of its crystalline structure within the composite.^47^ In addition, diffraction peaks located at approximately 30.88° and 43.33° were assigned to the (220) and (400) planes of cubic spinel Fe_3_O_4_ (magnetite), consistent with the standard reference pattern (PDF No. 01-089-2355).^48^ The characteristic (311) peak of Fe_3_O_4_ at around 35.4° was not clearly distinguishable due to overlap with the intense ZnO (101) reflection in the same region. The relatively low intensity and limited number of Fe_3_O_4_ reflections may be attributed to its lower phase content and/or reduced crystallinity within the composite matrix. Furthermore, slight shifts in peak positions compared with standard reference data were observed, which can be attributed to nanoscale effects, lattice strain, and interfacial interactions between ZnO and Fe_3_O_4_ phases.

#### 3.2.2. TEM, SEM-EDX and elemental mapping analysis of ZnO/Fe_3_O_4_ NCs

**Fig. 2:**
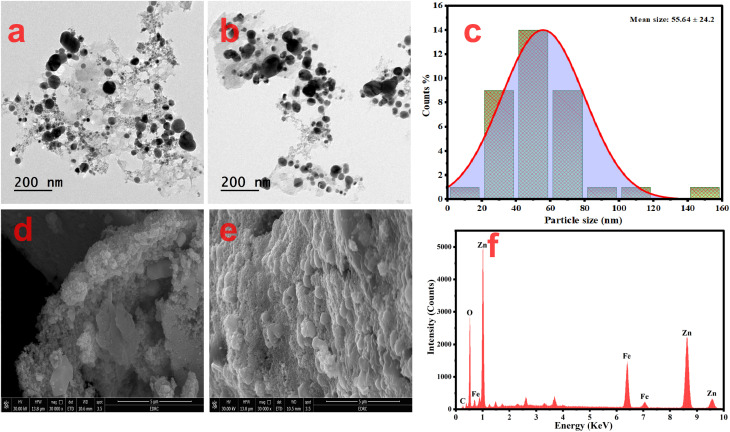
(a and b) TEM image, (c) PSA histogram of TEM (d and e) SEM image, and (f) EDX analysis of bimetallic ZnO/Fe_3_O_4_ NCs by tangerine peel extract.

The structural characteristics and particle size distribution of the green-synthesized ZnO/Fe_3_O_4_ NCs were investigated by TEM, as illustrated in Fig. 2a and b. The nanocomposites were fabricated using a natural extract derived from tangerine peels, which served as a bio-reducing and stabilizing agent during synthesis. TEM analysis provided detailed insight into particle morphology, dimensional uniformity, and dispersion behavior. The micrographs reveal that the prepared ZnO/Fe_3_O_4_ NCs predominantly exhibit a quasi-spherical to spherical morphology with relatively good distribution and moderate aggregation. The calculated average particle diameter was 55.64 ± 24.2 nm (Fig. 2c), indicating nanoscale dimensions suitable for enhanced surface reactivity and potential biological interactions.^49^ The relatively small size and high surface-to-volume ratio of these nanocomposites are factors that may contribute to their improved performance in biomedical and antimicrobial applications. Comparable findings have been reported in the literature. Hussein *et al.*^50^ described nanoparticles with a similar spherical morphology and particle sizes ranging from 50 to 90 nm based on TEM observations. Additionally, another study^51^ documented Fe–Zn nanoparticles with a spherical structure and particle diameters between 20 and 60 nm. The present results align well with these previous reports, further confirming that biosynthetic approaches can successfully produce nanocomposites with controlled morphology and nanoscale dimensions.

The surface morphology and structural features of the prepared nanocomposite were thoroughly investigated using SEM. The corresponding micrograph is presented in Fig. 2d and e. The SEM analysis revealed that the bimetallic ZnO/Fe_3_O_4_ NCs possesses a predominantly oval to irregular crystalline morphology with a noticeably porous texture. Such structural characteristics are commonly observed in metallic nanocomposites and can be attributed to their elevated surface energy, which promotes strong interparticle attraction and aggregation. The images further indicate that smaller primary nanoparticles tend to cluster and coalesce, forming larger secondary assemblies. This aggregation process leads to the development of a hybrid nanostructured framework, where individual nano units integrate into more complex architectures. The observed clustering behavior in the SEM micrographs reflects the early formation stages of a well-defined nanocomposite structure and confirms successful composite development.^50^ Comparable observations have been reported in previous studies. For instance, Alshehri and Malik^52^ demonstrated through SEM analysis that biosynthesized Cu–Co–Ni trimetallic nanoparticles exhibit pronounced structural porosity resulting from the agglomeration of nanoscale flakes. Likewise, another study^51^ described Fe–Zn nanoparticles with a spherical morphology and particle sizes ranging from 20 to 60 nm.

**Fig. 3:**
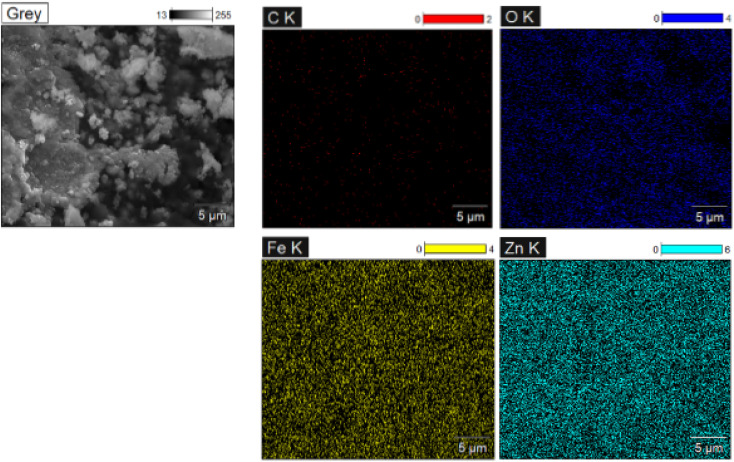
Elemental mapping of ZnO/Fe_3_O_4_ NCs showing uniform elemental distribution.

The elemental composition and distribution of the synthesized bimetallic ZnO/Fe_3_O_4_ NCs were examined using energy-dispersive X-ray (EDX) analysis coupled with elemental mapping (Fig. 2f). EDX is a reliable technique for confirming the presence of constituent elements and evaluating their spatial homogeneity within nanostructured materials, which is critical for correlating composition with functional properties.^53^ The EDX spectrum confirmed that the synthesized nanoparticles were mainly composed of iron (Fe), zinc (Zn), and oxygen (O). Quantitative analysis showed weight percentages of 16.3% Fe, 58.3% Zn, and 24.3% O, with corresponding atomic percentages of 10.4%, 31.9%, and 54.2%, respectively. The dominance of zinc with effective incorporation of iron confirms the presence of both Zn and Fe within the nanocomposite system, in agreement with previous reports on Fe–Zn bimetallic systems.^54^ Elemental mapping demonstrated a uniform and homogeneous distribution of Fe and Zn throughout the nanoparticle surface, indicating efficient interaction between the two metals and absence of phase segregation (Fig. 3). Such homogeneous elemental dispersion is known to enhance synergistic effects in bimetallic nanoparticles, leading to improved catalytic and biological activities.^55^ In addition to the main elements, minor carbon (C) signal was detected, these are likely derived from phytochemical residues of the plant extract used during green synthesis, which act as natural reducing and capping agents. The presence of these organic moieties may contribute to partial surface stabilization of the nanoparticles; however, some degree of aggregation was still evident from the DLS analysis.^56^

#### 3.2.3. DLS and zeta potential analysis of ZnO/Fe_3_O_4_ NCs

**Fig. 4:**
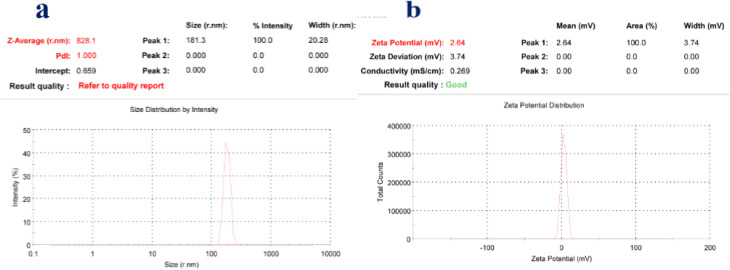
DLS-derived particle size distribution (a) and zeta potential profile (b) of the ZnO/Fe_3_O_4_ NCs.

Dynamic light scattering (DLS) analysis showed that the biosynthesized ZnO/Fe_3_O_4_ NCs exhibited a main hydrodynamic size peak at approximately 181.3 nm, with a *Z*-average diameter of 828.1 nm and a PDI of 1.000 (Fig. 4a). The larger hydrodynamic size compared with TEM results is attributed to nanoparticle agglomeration and the presence of a hydration layer and phytochemical capping agents from the tangerine peel extract, which commonly increase the apparent particle size in aqueous media.^18^ Such differences between DLS and TEM measurements are typical for green-synthesized nanoparticles and indicate aggregation and broad size distribution rather than poor size control.^57^ Zeta potential analysis revealed a low positive surface charge of +2.64 mV with a deviation of 3.74 mV (Fig. 4b), suggesting limited electrostatic colloidal stability.^58^ In plant-mediated nanoparticle systems, stability is often governed by steric effects provided by adsorbed biomolecules rather than strong electrostatic repulsion.^59^ Similar low zeta potential values have been reported for biosynthesized nanocomposites stabilized by phenolic and polysaccharide compounds.^60,61^ The DLS results revealed a relatively high polydispersity index and low zeta potential, indicating a tendency toward aggregation and limited colloidal stability. Such aggregation behavior may influence nanoparticle sedimentation, surface interactions with bacterial and cellular membranes, cellular uptake efficiency, and the reproducibility of biological assays. Therefore, the observed antibacterial and cytotoxic activities should be interpreted considering the dispersion state of the nanocomposites under the experimental conditions.

### 3.3. Antibacterial and molecular effects of ZnO/Fe_3_O_4_ nanocomposites on MDR *Acinetobacter baumannii*

#### 3.3.1. Antibiotic resistance profile of *A. baumannii* isolates

The antimicrobial susceptibility results of the fifteen *Acinetobacter baumannii* isolates are presented in Table S1. A high level of resistance was observed against most of the tested antibiotics, including trimethoprim/sulfamethoxazole, ciprofloxacin, levofloxacin, cefotaxime, ceftazidime, piperacillin/tazobactam, and imipenem. Based on the standard definition of multidrug resistance (MDR), defined as resistance to at least three different classes of antimicrobial agents, four isolates (A, B, C, and D) were classified as multidrug-resistant (MDR) *A. baumannii*. These isolates exhibited resistance to multiple antibiotic classes, including fluoroquinolones, β-lactams, aminoglycosides, carbapenems, and sulfonamides. The remaining isolates demonstrated varying resistance patterns but did not fulfill the full criteria for MDR classification according to the adopted definition.

#### 3.3.2. Antibacterial activity of ZnO/Fe_3_O_4_ nanocomposites

**Fig. 5:**
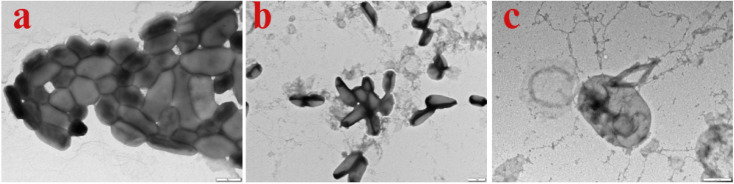
Transmission electron microscopy micrographs of control (a) and ZnO/Fe_3_O_4_ NCs-treated *Acinetobacter baumannii* (b and c).

The antibacterial activity of ZnO/Fe_3_O_4_ NCs against four multidrug-resistant (MDR) *Acinetobacter baumannii* isolates (A, B, C, and D) demonstrated clear inhibitory effects, as evidenced by measurable inhibition zones in the agar well diffusion assay. The inhibition zone diameters ranged from approximately 17 mm to 24 mm (Fig. S4 and S5). Despite their resistance to multiple antibiotic classes, all MDR isolates were measurable inhibitory activity in response to the nanocomposites. These findings suggest that ZnO/Fe_3_O_4_ NCs represent a hopeful alternative strategy for combating MDR *A. baumannii* infections.^62^ To experimentally evaluate their antimicrobial efficacy, the MIC was determined using the standard broth microdilution assay conducted in sterile 96-well microplates. The MIC, defined as the lowest nanocomposite concentration capable of completely suppressing visible bacterial growth, was identified as 500 µg mL^−1^ for multidrug-resistant *Acinetobacter baumannii* isolates A and B. In contrast, isolates C and D exhibited greater susceptibility, with complete growth inhibition observed at 250 µg mL^−1^. Moreover, the sub-inhibitory concentration (sub-MIC), which represents the concentration range below the MIC that does not fully inhibit growth but may influence bacterial behavior, was found to lie between 125 and 250 µg mL^−1^. The observed MIC values (250–500 µg mL^−1^) indicate moderate antibacterial activity compared to some previously reported nanomaterials, which may exhibit lower MIC values.^63^ The observed antibacterial activity may be related to physicochemical interactions between ZnO/Fe_3_O_4_ NCs and bacterial cells under the experimental conditions. The nanoscale particle size observed by TEM may facilitate surface interactions with bacterial membranes, whereas the aggregation behavior and limited colloidal stability indicated by DLS and zeta potential analyses may influence nanoparticle dispersion and contact under biological conditions.^64,65^ In addition to the MIC assessment, TEM was employed to investigate the nanoscale structural alterations (ultrastructural changes) induced by ZnO/Fe_3_O_4_ NCs in bacterial cells (Fig. 5). Ultrastructure refers to cellular features observed at the nanometer level, including membrane integrity and intracellular organization, as visualized by TEM. The control (Fig. 5a) displayed intact cells with well-preserved cell envelopes and homogeneous cytoplasmic content, indicating normal cellular morphology and structural integrity. In contrast, bacteria treated with ZnO/Fe_3_O_4_ NCs (Fig. 5b and c) exhibited pronounced morphological and ultrastructural alterations. These changes included deformation and shrinkage of cells, disruption of the outer membrane, irregular thickening or partial detachment of the cell wall, and condensation or leakage of cytoplasmic material. In some cells, marked membrane damage and loss of intracellular organization were observed, suggesting irreversible cellular damage. The observed structural deterioration is consistent with the proposed antibacterial mechanisms of ZnO/Fe_3_O_4_ NCs, which involve ROS generation, direct nanoparticle–cell membrane interactions, and disruption of membrane permeability and integrity. The close association of nanocomposite aggregates with the bacterial surface further supports the hypothesis of physical contact-mediated damage in addition to oxidative stress.^66^

#### 3.3.3. Impact of ZnO/Fe_3_O_4_ NCs upon CRISPR-associated gene expression in multidrug-resistant *Acinetobacter baumannii*

**Fig. 6:**
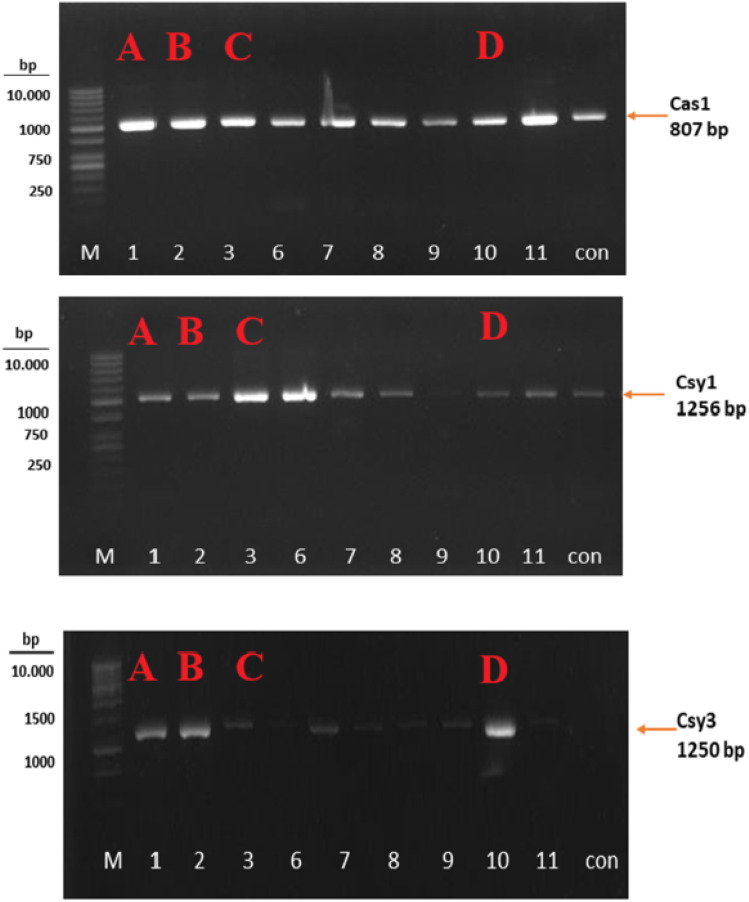
PCR amplification of CRISPR-associated genes (Cas1, Csy1, and Csy3) in *Acinetobacter baumannii* isolates. The target genes were screened in nine multidrug-resistant clinical isolates in addition to a sensitive control isolate (lanes 1–11). Among the screened isolates, four multidrug-resistant isolates harboring the target genes were selected and designated as A, B, C, and D for subsequent analyses. Red arrows indicate the amplified target genes. Lane M: DNA ladder.

**Fig. 7:**
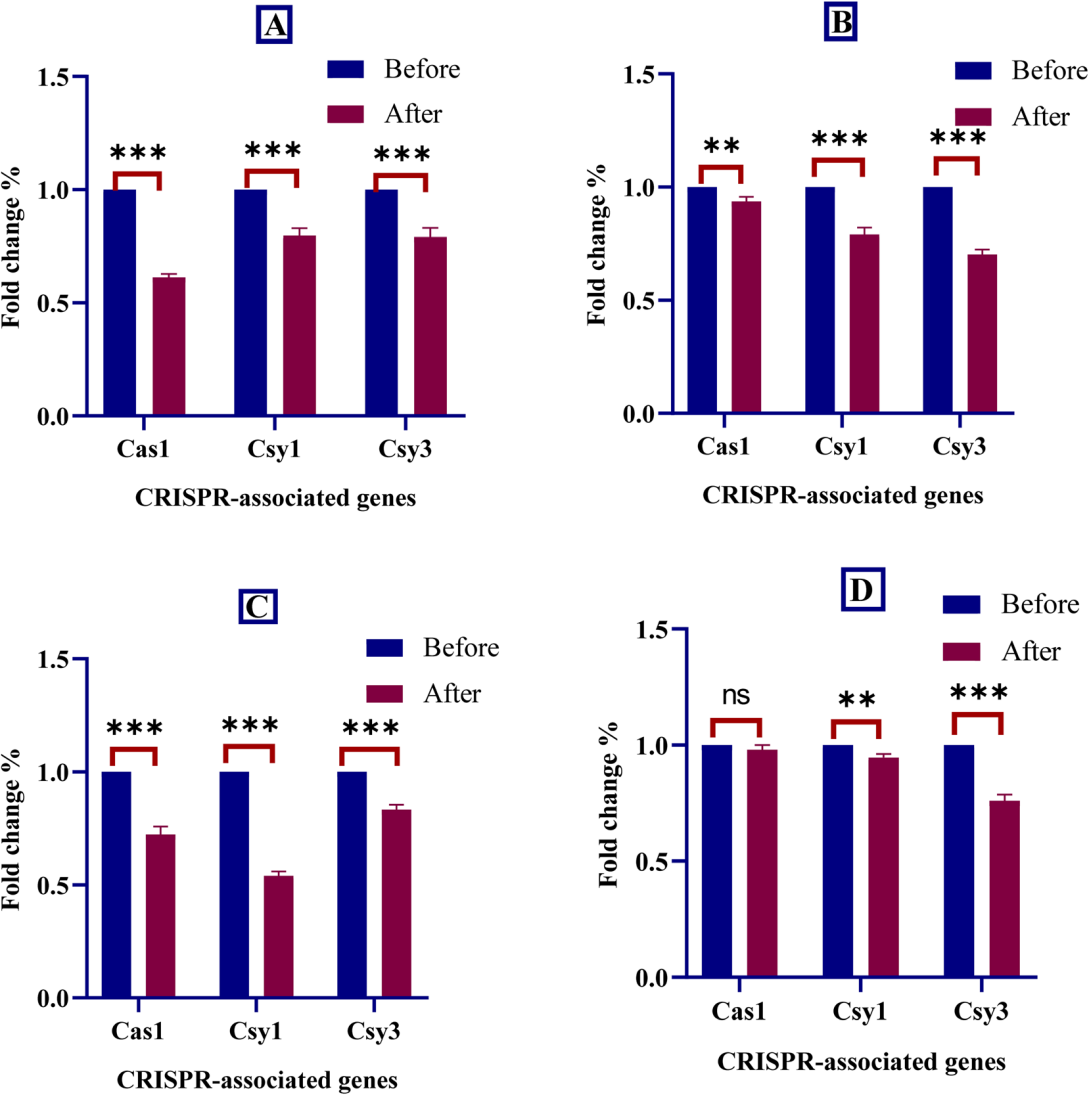
Effect of sub-MIC concentrations of ZnO/Fe_3_O_4_ NCs on the expression of CRISPR-associated genes (Cas1, Csy1, and Csy3) in multidrug-resistant *Acinetobacter baumannii* isolates (A–D). Data are presented as mean ± standard deviation (SD) from three independent experiments. Statistical analysis was performed using two-way ANOVA followed by Tukey's post hoc test. Statistical significance is indicated as follows: ns (not significant), *p < 0.05, **p < 0.01, and ***p < 0.001.

The notable antibacterial activity of ZnO/Fe_3_O_4_ NCs can be attributed to their unique structural and surface-related features. Their high surface area, reactive sites, surface charge interactions, and potential to generate reactive oxygen species collectively enhance their ability to interact with and disrupt bacterial cell membranes. These combined physicochemical attributes contribute to membrane damage, oxidative stress induction, and ultimately bacterial cell death, highlighting the potential role of ZnO/Fe_3_O_4_ NCs as antimicrobial nanomaterials. The present study evaluated the effect of sub-MIC concentrations of ZnO/Fe_3_O_4_ NCs on the relative expression of CRISPR-associated genes (Cas1, Csy1, and Csy3) in four multidrug-resistant *Acinetobacter baumannii* isolates (A–D) (Fig. 6). The results revealed a consistent downregulation of all three genes following treatment compared to untreated controls (Fig. 7). The expression levels of Cas1 ranged from 0.61 to 0.98, with the lowest expression observed in isolate A, while isolates B and D exhibited values closer to the control. Similarly, Csy1 expression showed a marked reduction, particularly in isolate C (0.54), indicating a pronounced sensitivity of this gene to ZnO/Fe_3_O_4_ NC exposure. In contrast, isolates A, B, and D demonstrated moderate decreases in Csy1 expression. The expression of Csy3 also declined across all isolates, with values ranging between 0.70 and 0.83, suggesting isolate-dependent variability in response to nanocomposite treatment. The observed downregulation of CRISPR-associated genes may be associated with a general stress response induced by ZnO/Fe_3_O_4_ NCs at sub-inhibitory concentrations. Metal oxide nanocomposites are known to induce oxidative stress, disrupt membrane integrity, and interfere with intracellular processes, which can lead to global transcriptional changes. Recent studies have suggested that nanoparticle-induced stress may influence bacterial defense-related pathways, including CRISPR-Cas systems, which are energetically demanding and tightly regulated.^67^ Cas1 plays a critical role in spacer acquisition and CRISPR adaptation, and its reduced expression may indicate a potential impact on adaptive responses. Likewise, Csy1 and Csy3, essential components of the type I–F CRISPR interference complex, exhibited decreased expression, which may reflect alterations in gene regulation under stress conditions.^68^ This observation may suggest a potential increase in bacterial susceptibility to stress conditions; however, this interpretation requires further functional validation.^69^ However, it should be noted that gene expression changes alone do not confirm functional inhibition of the CRISPR-Cas system, and further functional or genetic studies are required to validate this effect. These findings suggest that ZnO/Fe_3_O_4_ NCs exert antibacterial activity not only through direct physicochemical interactions but also may be associated with modulation of bacterial gene expression. The observed downregulation of CRISPR-associated genes should therefore be interpreted cautiously as a transcriptional response rather than direct evidence of impaired CRISPR-Cas functionality, but it may provide preliminary insight into the interaction between nanomaterials and bacterial adaptive systems.

### 3.4. Cytotoxic and antiproliferative effects of ZnO/Fe_3_O_4_ NCs on Vero and Caco-2 cells

#### 3.4.1. MTT assay

**Fig. 8:**
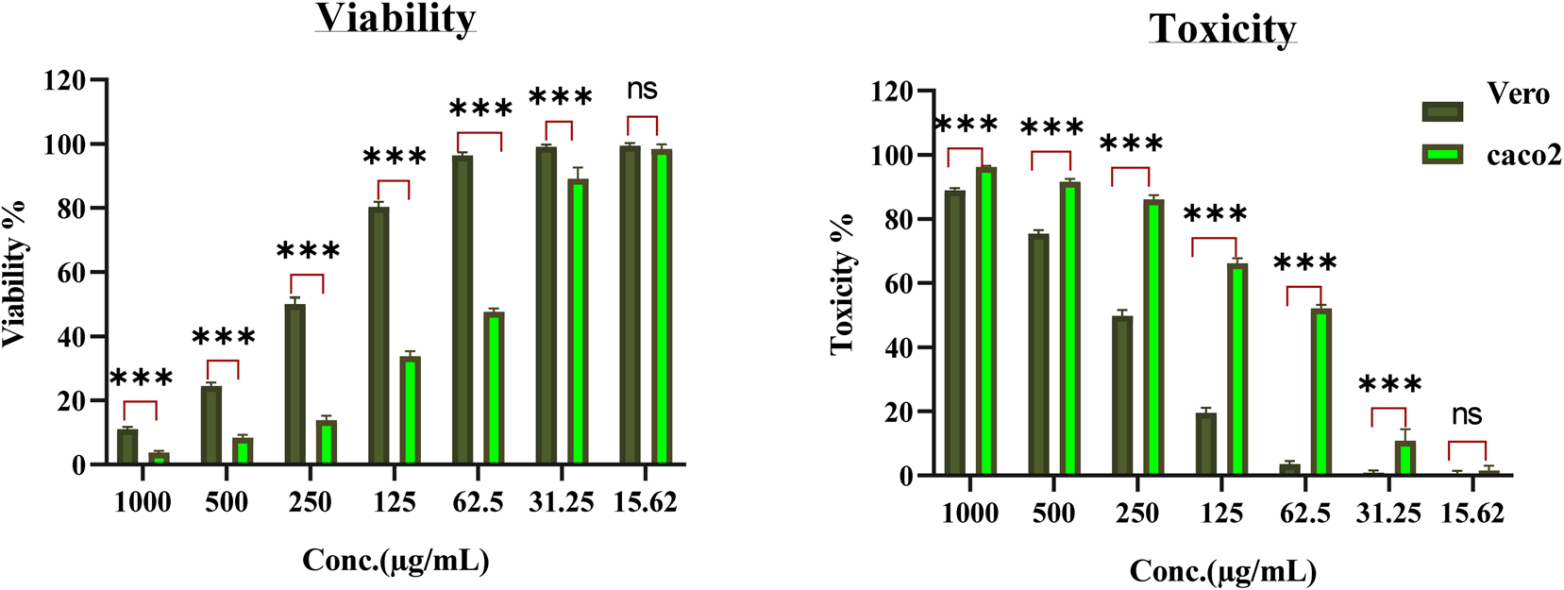
Effect of ZnO/Fe_3_O_4_ NCs on cell viability and toxicity of Vero and Caco-2 cells at different concentrations (µg mL^−1^). Data are presented as mean ± standard deviation (SD) from three independent experiments. Statistical analysis was performed using two-way ANOVA followed by Tukey's post hoc test. Statistical significance is indicated as ns (not significant), and ***p < 0.001.

**Fig. 9:**
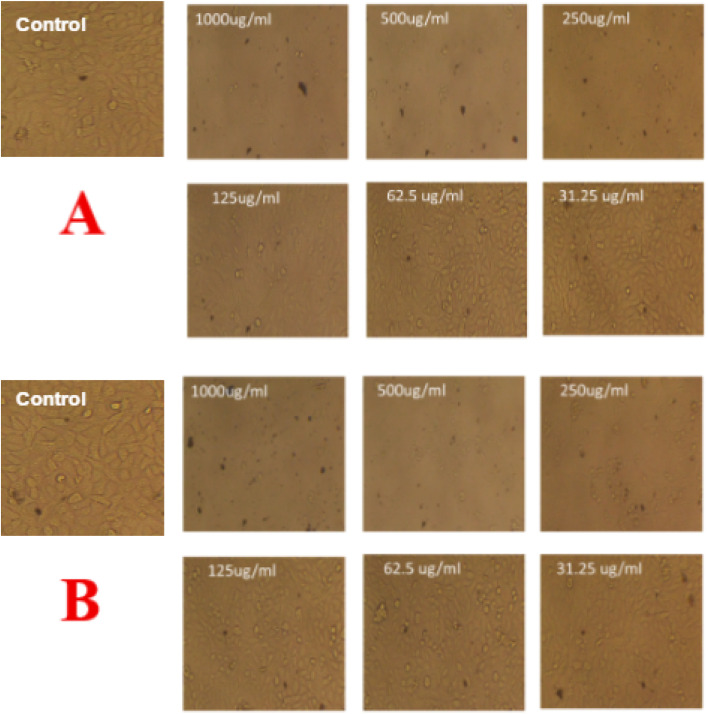
Morphological changes of (A) Vero and (B) Caco-2 cells after treatment with different concentrations of ZnO/Fe_3_O_4_ NCs.

The cytotoxic potential of the ZnO/Fe_3_O_4_ NCs was evaluated against Vero and Caco-2 cell lines at various concentrations ranging from 1000 to 15.62 µg mL^−1^ (Fig. 8). The results demonstrated a dose-dependent reduction in cell viability for both cell types, with the inhibitory effect increasing as the concentration of the nanocomposites increased. At higher concentrations (1000 and 500 µg mL^−1^), Vero cells showed viabilities of 88.97% and 75.47%, respectively, while Caco-2 cells exhibited viabilities of 96.22% and 91.63%. As the concentration decreased, a marked reduction in cell viability was observed, particularly in Vero cells. At 62.5 µg mL^−1^, viability decreased to 3.52% in Vero cells compared to 52.30% in Caco-2 cells. The calculated IC_50_ values further confirmed the higher sensitivity of Caco-2 cells toward ZnO/Fe_3_O_4_ NCs, with an IC_50_ of 60.7 µg mL^−1^ compared to 254.66 µg mL^−1^ for Vero cells, indicating selective cytotoxicity toward cancer cells under the tested conditions. These results suggest that ZnO/Fe_3_O_4_ NCs exhibit measurable and concentration-dependent cytotoxic effects, with relatively lower toxicity toward normal Vero cells. Such selectivity is considered a favorable feature for potential biomedical applications.^70^ The enhanced sensitivity of Caco-2 cells may be related to differences in cellular characteristics, such as metabolic activity or susceptibility to stress conditions, although this was not directly investigated in the present study.^71^ Previous studies have reported that ZnO-based nanomaterials can be associated with oxidative stress and apoptotic responses in cancer cells,^72^ and the presence of Fe_3_O_4_ may influence cellular interactions and uptake.^73^ However, the current study does not directly evaluate these mechanisms, and therefore such interpretations should be considered as possible explanations rather than confirmed pathways. Similarly, earlier reports have demonstrated that ZnO- and Fe_3_O_4_-based nanocomposites can show selective cytotoxicity toward cancer cells,^74,75^ which is consistent with the trends observed in this study. Moreover, the higher IC_50_ value in Vero cells suggests comparatively lower toxicity toward normal cells, aligning with previously reported biocompatibility profiles of similar nanomaterials.^76,77^ Microscopic examination revealed distinct morphological alterations in both cell lines following treatment with ZnO/Fe_3_O_4_ NCs (Fig. 9). Untreated Vero and Caco-2 cells exhibited normal morphology with intact membranes and typical cellular architecture. In contrast, treated cells showed concentration-dependent morphological changes, including cell shrinkage, loss of adhesion, membrane blebbing, and reduced cell density.

#### 3.4.2. Effects of ZnO/Fe_3_O_4_ NCs on apoptosis and cell cycle progression in Caco-2 cells

**Fig. 10:**
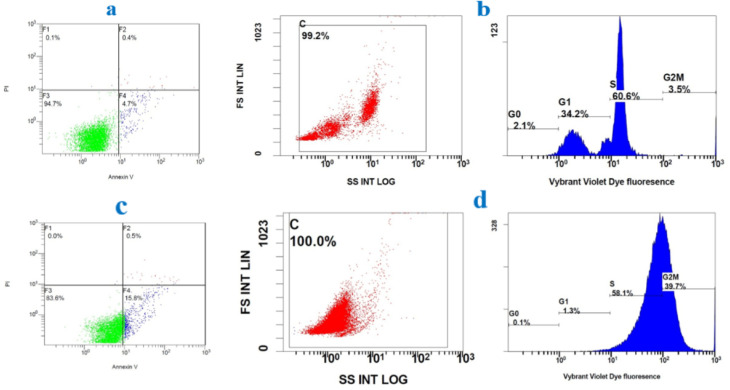
Flow cytometry assessment of apoptotic activity and cell cycle progression in Caco-2 cells. Panels (a) and (b) illustrate the baseline apoptotic profile and cell cycle phase distribution of untreated control cells, showing the normal pattern of viable, early apoptotic, late apoptotic, and necrotic populations, along with the typical distribution across G0/G1, S, and G2/M phases. In contrast, panels (c) and (d) present the corresponding analysis for cells exposed to ZnO/Fe_3_O_4_ NCs, highlighting the treatment-induced alterations in apoptotic cell percentages and modifications in cell cycle progression. The comparative results demonstrate the impact of the nanocomposite exposure on cellular viability, apoptosis induction, and potential cell cycle arrest in Caco-2 cells.

Flow cytometric analysis was conducted to investigate apoptosis and cell cycle distribution in untreated and ZnO/Fe_3_O_4_ NCs -treated Caco-2 cells. As shown in Fig. 10 and Table S4, untreated cells exhibited a high viability rate (94.7%) with minimal early apoptosis (4.7%), followed by late apoptosis (0.4%) and necrosis (0.1%). Upon treatment with ZnO/Fe_3_O_4_ NCs, there was a decline in cell viability (83.6%), accompanied by an increase in early apoptotic cells (15.8%). Late apoptosis remained limited (0.5%), and necrotic cells were not detected, suggesting that the nanocomposites primarily induce apoptotic rather than necrotic cell death under the tested conditions, which is consistent with previous findings on ZnO-based nanomaterials.^78,79^ Cell cycle analysis further revealed alterations in treated cells. While untreated Caco-2 cells were mainly distributed in the S phase (60.6%) and G1 phase (34.2%), treated cells exhibited an accumulation in the G2/M phase (39.7%), along with a reduction in G1 (1.3%) and G0 (0.1%) phases. This pattern is consistent with cell cycle arrest at the G2/M phase, which has been reported for ZnO-containing nanomaterials.^80^ The present results demonstrate that ZnO/Fe_3_O_4_ NCs are associated with increased apoptotic cell populations and alterations in cell cycle distribution in Caco-2 cells. The increase in early apoptotic cells, with negligible necrosis, suggests that programmed cell death may be a dominant response under these experimental conditions.^81^ The observed accumulation of cells in the G2/M phase may indicate an effect on cell cycle progression, although the underlying molecular mechanisms were not investigated in the present study. Previous studies have suggested that ZnO-based nanomaterials can be associated with oxidative stress and DNA damage responses, which may contribute to such effects.^82^ However, these mechanisms were not directly evaluated here and should therefore be interpreted as possible explanations rather than confirmed pathways. Similarly, reports have indicated that ZnO/Fe_3_O_4_ NCs may influence intracellular processes related to apoptosis,^83^ but further studies are required to confirm the involvement of specific pathways such as ROS generation, mitochondrial dysfunction, or caspase activation in this system. The biological performance of ZnO/Fe_3_O_4_ NCs can be correlated with their physicochemical properties. The nanoscale size observed by TEM may facilitate interaction with bacterial cell membranes and enhance cellular uptake.^84^ However, the relatively large hydrodynamic size and high polydispersity index obtained from DLS indicate aggregation, which may reduce dispersion stability and affect reproducibility.^85^ The low zeta potential further supports limited colloidal stability, potentially influencing surface interactions.^86^ In addition, the coexistence of ZnO and Fe_3_O_4_ phases may contribute to synergistic effects, enhancing antibacterial and cytotoxic responses through combined physicochemical mechanisms.^87^

### 3.5. Evaluation of the antioxidant activity of ZnO/Fe_3_O_4_ NCs

**Fig. 11:**
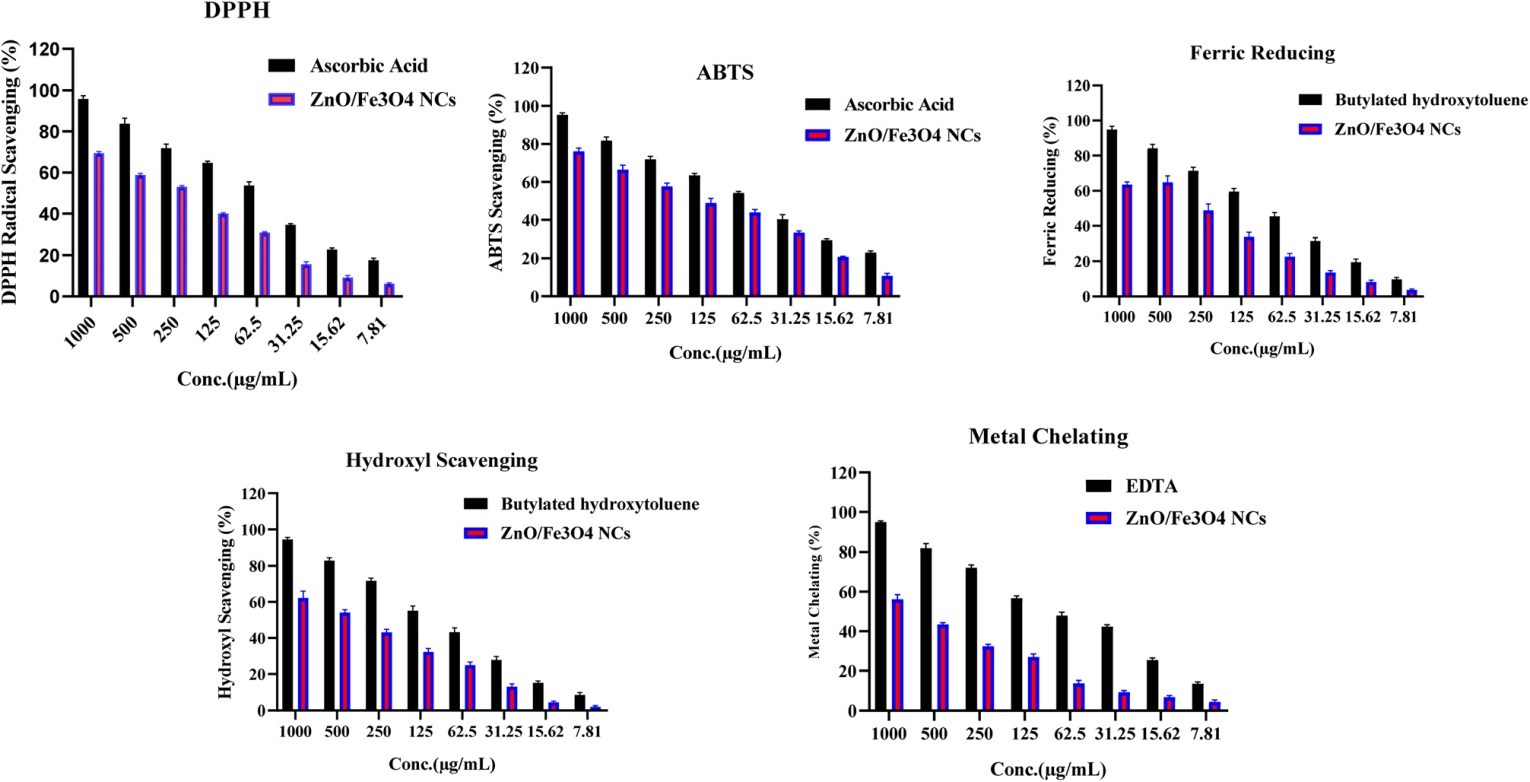
Antioxidant activity of ZnO/Fe_3_O_4_ NCs compared with standard antioxidants (ascorbic acid, butylated hydroxytoluene, and EDTA) using DPPH, ABTS, ferric reducing power, hydroxyl radical scavenging, and metal chelating assays at different concentrations (µg mL^−1^). Data are presented as mean ± SD from three independent experiments. Statistical analysis was performed using two-way ANOVA followed by Tukey's post hoc test. Statistical comparisons were conducted between ZnO/Fe_3_O_4_ NCs and the corresponding standard at each corresponding concentration. Statistical significance was ***p < 0.001 for all measurements, except at 7.8 µg mL^−1^ where *p < 0.05 was observed in the ferric reducing power assay and **p < 0.01 in the hydroxyl radical scavenging assay.

The antioxidant activity of ZnO/Fe_3_O_4_ NCs was evaluated using DPPH, ABTS, hydrogen peroxide (H_2_O_2_) scavenging, ferric reducing and metal chelating assays (Fig. 11). The antioxidant assays were performed to provide complementary information on the redox-related properties of the nanocomposites. The nanocomposites exhibited a clear concentration-dependent antioxidant behavior across all tested systems, which is a common characteristic of metal oxide-based nanomaterials.^88^ In the DPPH assay, ZnO/Fe_3_O_4_ NCs had been a gradual elevation in radical scavenging activity in relatively to the quantities, reaching 69.44% at 1000 µg mL^−1^. Nevertheless, the scavenging activity was consistently little effect than that of the standard antioxidants, ascorbic acid which showed inhibition values above 94%. Similar trends have been reported for ZnO-containing nanocomposites, where moderate DPPH scavenging activity was observed compared to conventional antioxidants.^89^ The ABTS assay revealed higher scavenging percentages compared to DPPH, with ZnO/Fe_3_O_4_ NCs achieving up to 76.12% inhibition at the highest concentration. This enhanced response can be attributed to the greater sensitivity of ABTS radicals toward both hydrophilic and lipophilic antioxidant systems.^90^ In the H_2_O_2_ scavenging assay, ZnO/Fe_3_O_4_ NCs exhibited moderate activity, reaching 62.24% inhibition at 1000 µg mL^−1^, while BHT showed significantly higher scavenging efficiency. This dose-dependent behavior is consistent with previous reports on magnetic metal oxide nanostructures.^75^ Metal chelating results indicated that ZnO/Fe_3_O_4_ NCs possess appreciable chelating ability, with a maximum of 56.13% at 1000 µg mL^−1^. Although EDTA remained the most effective chelator, the observed activity of the nanocomposites aligns with studies highlighting the role of Fe_3_O_4_ surfaces in binding transition metal ions.^91^ The reducing power of ZnO/Fe_3_O_4_ NCs was evaluated and compared with the standard antioxidant butylated hydroxytoluene. At the highest concentration (1000 µg mL^−1^), ZnO/Fe_3_O_4_ NCs exhibited a reducing power of 63.52%, while BHT showed a significantly higher value of 94.86%. The present findings confirm that ZnO/Fe_3_O_4_ NCs indicate moderate antioxidant activity through free radical scavenging, hydrogen peroxide neutralization, reducing power and metal ion chelation mechanisms. The concentration-dependent patterns observed across all assays reflect the increasing availability of active surface sites at higher concentrations, a phenomenon widely reported for metal oxide nanomaterials.^92^ The higher antioxidant values obtained in the ABTS assay compared to DPPH are in agreement with previous studies indicating that ABTS radicals are more responsive to nanomaterial-based antioxidants.^93^ This suggests that ZnO/Fe_3_O_4_ NCs may interact more efficiently with charged radical species. Although the antioxidant activity of ZnO/Fe_3_O_4_ NCs was lower than that of standard antioxidants, their moderate scavenging efficiency remains biologically relevant. Unlike small-molecule antioxidants, nanocomposites can provide sustained antioxidant effects due to their high surface area and structural stability.^94^ The antioxidant performance of ZnO/Fe_3_O_4_ NCs is comparable to previously reported ZnO- and Fe_3_O_4_-based nanomaterials, reinforcing their potential application as multifunctional agents in biomedical and pharmaceutical fields.^95^ However, these assays do not provide direct mechanistic insight into the biological effects and should be interpreted as supportive rather than explanatory.

## 4 Conclusion

In conclusion, the present study provides preliminary insights into green-synthesized ZnO/Fe_3_O_4_ NCs derived from *Citrus reticulata* peel extract as multifunctional nanomaterials with potential biomedical relevance. The eco-friendly synthesis approach, supported by the phytochemical richness of the peel extract, enabled the formation of structurally stable nanocomposites with measurable antibacterial, cytotoxic, and antioxidant activities. The observed antibacterial effect against multidrug-resistant *Acinetobacter baumannii*, along with ultrastructural cellular damage and downregulation of CRISPR-associated genes, suggests possible contributions from both physicochemical interactions and transcriptional responses, rather than confirming specific molecular mechanisms. Additionally, the selective cytotoxicity toward cancer cells and the induction of apoptosis and cell cycle alterations indicate potential anticancer effects under the tested conditions.

However, several limitations of the current study should be acknowledged. The relatively low zeta potential and high polydispersity index indicate possible nanoparticle aggregation, which may affect stability and reproducibility in biological systems. Furthermore, the mechanistic interpretation of CRISPR-Cas gene modulation remains preliminary and requires further functional validation. The biological evaluations were limited to *in vitro* experiments, and the absence of *in vivo* studies restricts the direct translation of these findings. In addition, the high-temperature drying step may have reduced the contribution of surface-bound phytochemicals, potentially influencing the observed bioactivity.

Future studies should focus on optimizing synthesis conditions to improve colloidal stability and dispersion behavior of the nanocomposites. Detailed mechanistic investigations, including reactive oxygen species generation, mitochondrial dysfunction, and molecular signaling pathways, are necessary to better understand the observed biological effects. Moreover, *in vivo* studies are essential to evaluate therapeutic efficacy, pharmacokinetics, and biosafety profiles. Functional assays to validate the role of CRISPR-Cas modulation in bacterial response to nanocomposite exposure are also recommended.

## Ethical approval

The study protocol was approved by the Research Ethics Committee, College of Science for Women, University of Baghdad (Ref: CSW-REC 1022; 12 June 2025). All procedures were performed in accordance with institutional and international ethical standards. Informed consent was obtained from all participants.

## Author contributions

N. M. A., M. R. M. and H. A. H.: writing original draft preparation, visualization, data curation and software. A. S. A. and K. M. A.: project administration, resources and funding acquisition. R. K. Z., H. H., L. M. A., N. T. A., and A. T. A.: experimental work, data curation, and writing—original draft preparation. M. A. K., A. S. B., and A. A.: methodology, investigation, data curation, visualization, and writing original draft preparation. M. K. Y. S.: conceptualization, supervision, visualization, formal analysis. All authors have read and agreed to the published version of the manuscript.

## Conflicts of interest

The authors declare no conflicts of interest.

## Supplementary Material

RA-016-D6RA02362B-s001

## Data Availability

The data sets used and/or analyzed during the current study available from the corresponding author on reasonable request. Supplementary information (SI) is available. See DOI: https://doi.org/10.1039/d6ra02362b.
