# Supplementary material for: Green synthesis of ZnO/Fe3O4 nanocomposites from Citrus reticulata peel: antibacterial activity against MDR Acinetobacter baumannii, CRISPR-Cas gene modulation, and anticancer potential
Source: RSC Adv. 2026 Jul 2;16(34):32990–3010. doi: 10.1039/d6ra02362b (PMC13326584; doi:10.1039/d6ra02362b)
Supplement: RA-016-D6RA02362B-s001 [file RA-016-D6RA02362B-s001.pdf]

Supplementary Information (SI) for RSC Advances.
This journal is © The Royal Society of Chemistry 2026

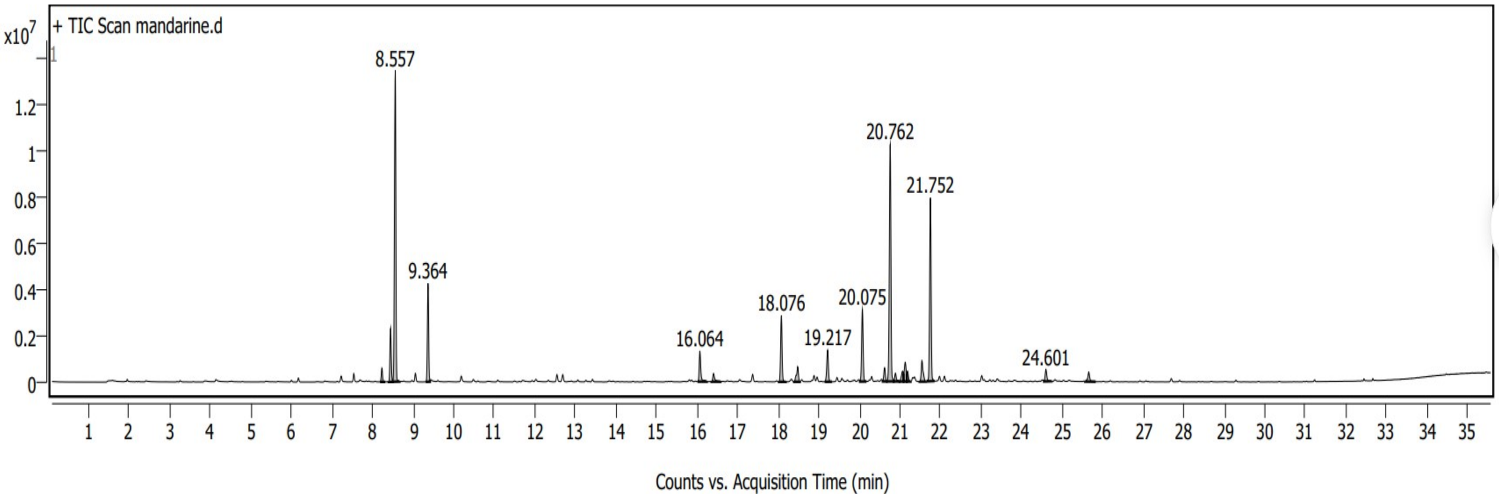

Fig. S1 GC-MS analysis of the Tangerine Peel Extract

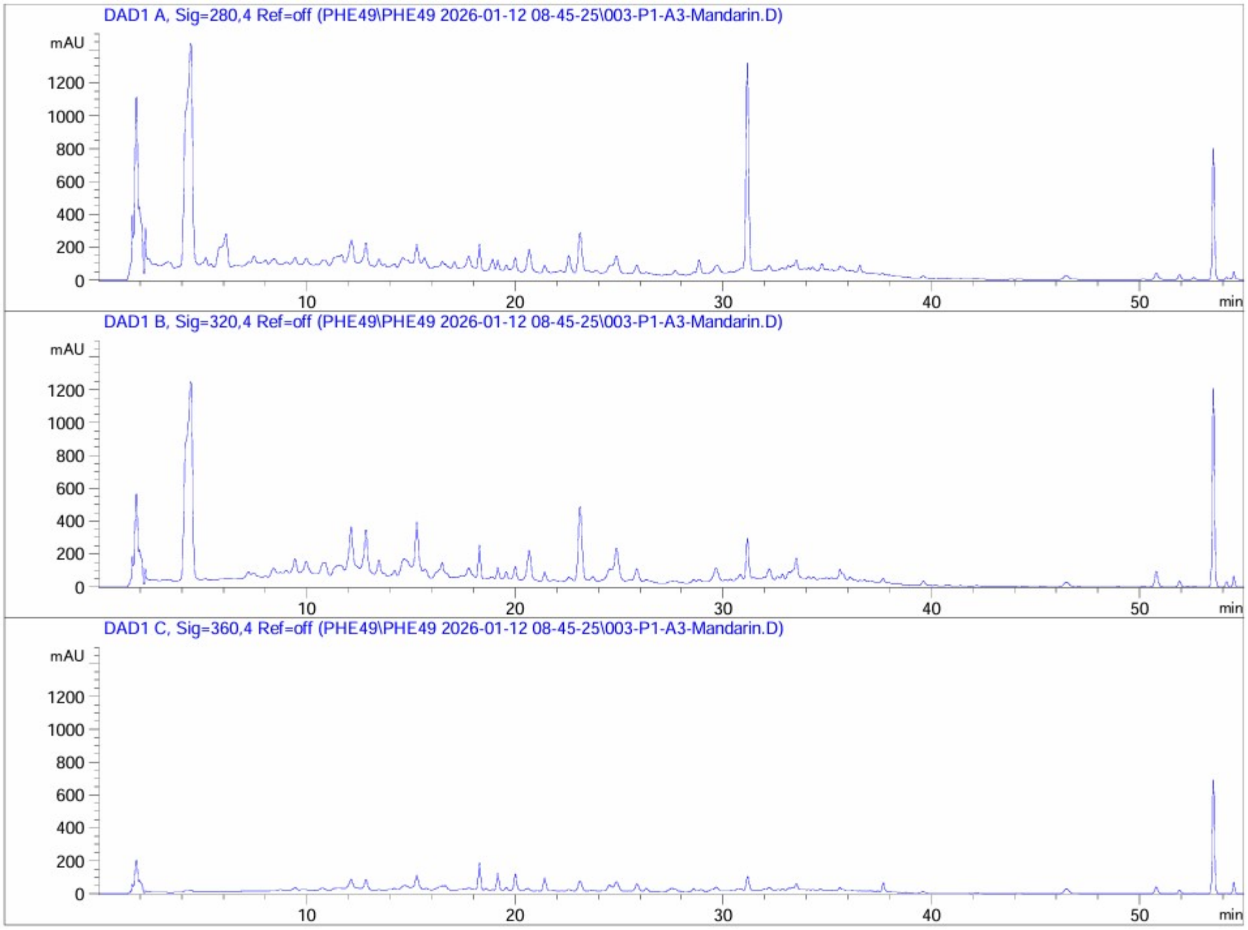

Fig. S2 Representative HPLC-DAD chromatograms showing phenolic and flavonoid constituents of *Citrus reticulata* peel extract

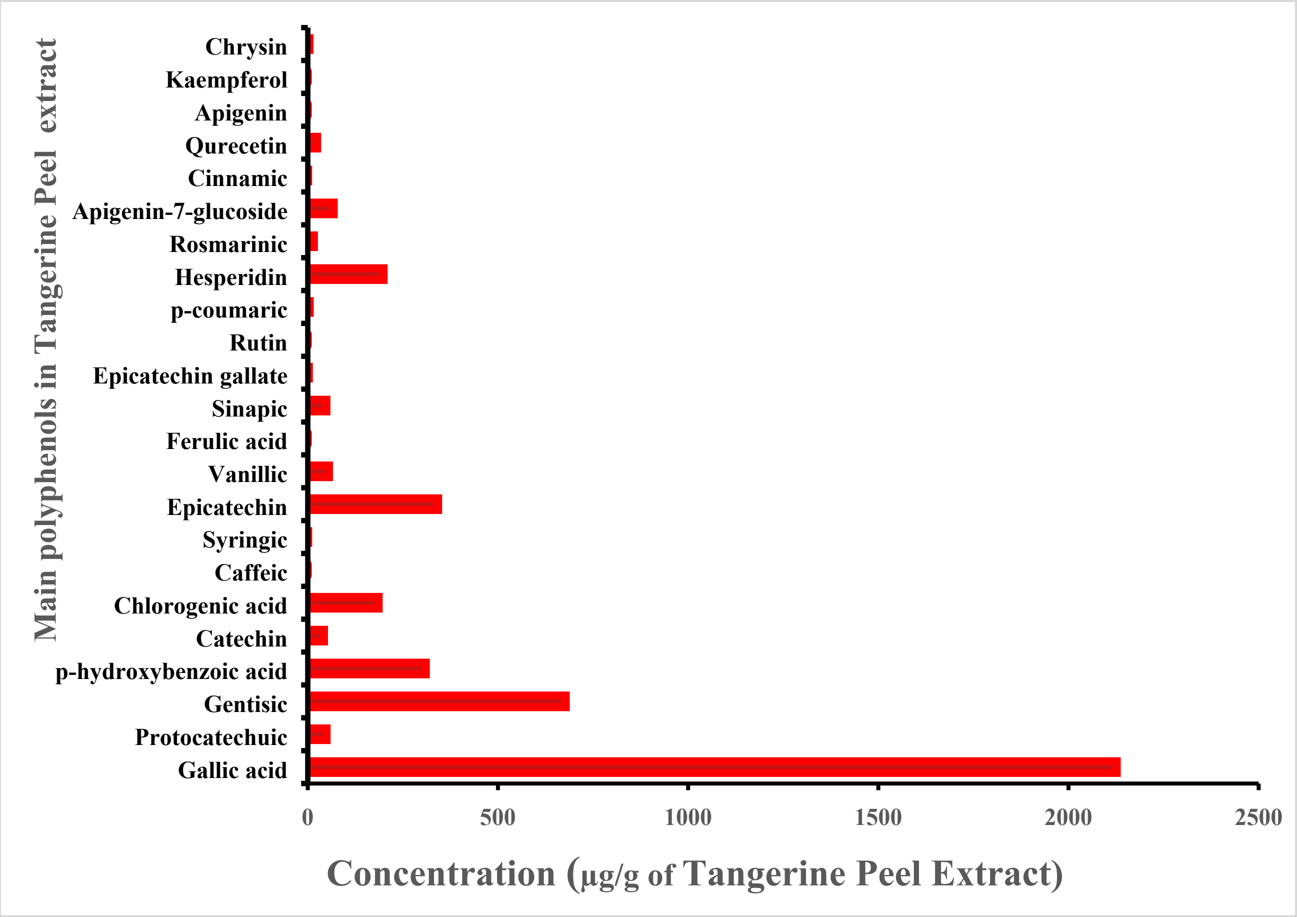

Fig. S3 HPLC analysis of major polyphenolic compounds in Tangerine peel extract

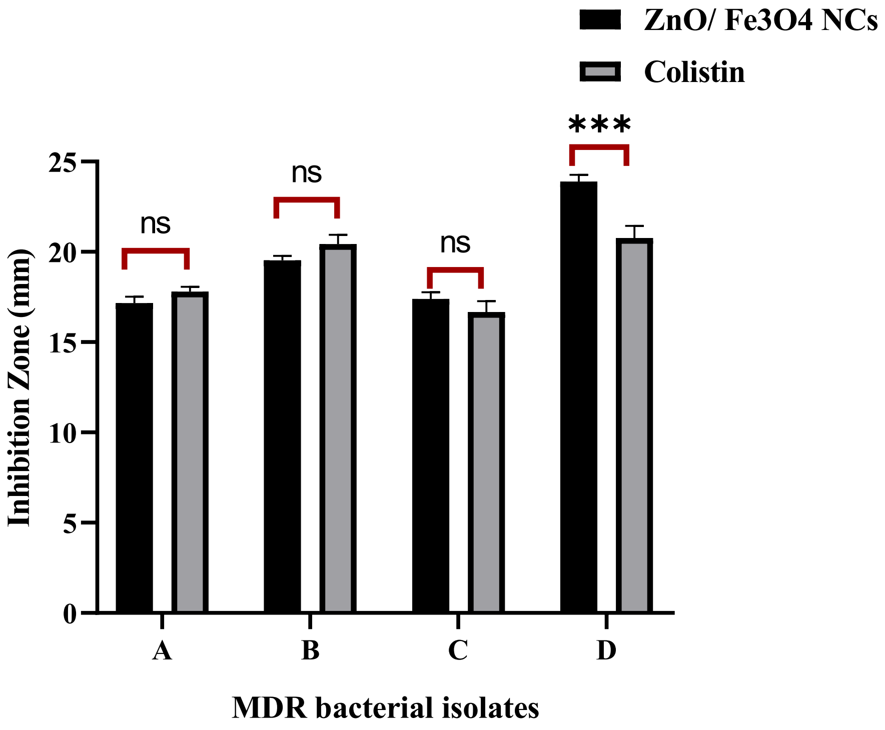

Fig. S4 Antibacterial activity of ZnO/Fe_3O_4 nanocomposites (NCs) compared with colistin against multidrug-resistant *Acinetobacter baumannii* isolates (A–D), expressed as inhibition zone diameter (mm). Data are presented as mean ± standard deviation (SD) from three independent experiments. Statistical analysis was performed using two-way ANOVA followed by Tukey's post hoc test. Statistical significance is indicated as ns (not significant) and ***$p < 0.001$.

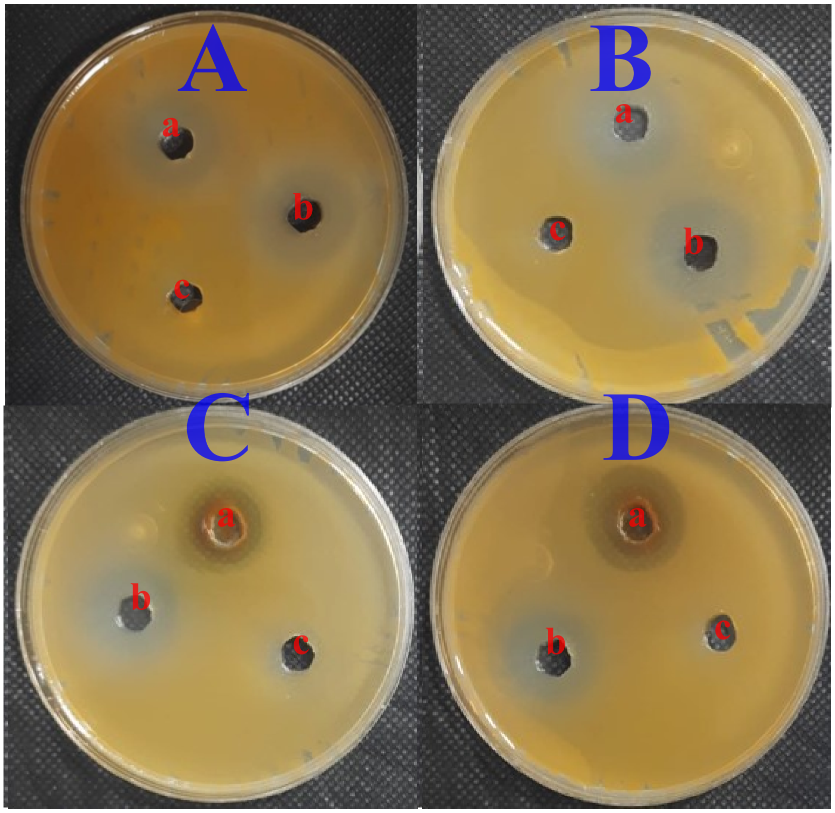

Fig. S5 Antibacterial activity of ZnO/Fe_3O_4 NCs against MDR *A. baumannii* using agar well diffusion. (a) NCs, (b) colistin (positive control), and (c) plant extract.

Table S1: Drug resistance profile of *A. baumanni* isolates

**No isolates**	**trimethoprim/ Sulfamethoxazole**	**CIP-10**	**LEV -5**	**C+O-10**	**CTX-10**	**CAZ-30**	**AK-10**	**GEN-10**	**TOB -10**	**P+L-100**	**IPM-10**	**DO-10**
**A**	+	+	+	+	+	+	+	+	+	+	+	+
**B**	+	+	+	+	+	+	+	+	+	+	+	+
**C**	+	+	+	+	+	+	+	+	+	+	+	+
**E**	+	+	+	-	+	+	+	+	+	+	+	+
**G**	+	+	+	+	+	+	+	+	+	+	+	+
**H**	+	+	+	+	+	+	+	+	+	+	+	+
**P**	+	+	+	+	+	+	-	+	+	+	+	-
**D**	+	+	+	+	+	+	+	+	+	+	+	+
**K**	+	+	+	+	+	+	+	+	+	+	+	+
**L**	+	+	+	+	+	+	+	+	+	+	+	+
**O**	+	-	+	+	+	+	+	+	+	+	+	+
**N**	+	+	+	+	+	+	+	+	+	+	+	+
**M**	+	+	+	+	+	+	+	+	+	+	+	-
**X**	+	+	+	+	+	+	-	+	+	+	+	+
**Z**	+	+	+	+	+	+	+	+	+	+	+	-

Table S2: The list of primers used in the CRISPR genes detection. All primer sequences are in 5'-3' orientation.

Genes	Sequences (5′-3′)	Length (bp)
IFb-cas1-F	AAGCTGCGATGCGAATGTTATGTA	807

IFb-cas1-R	CTATGGCTAATTCCTAGTACCCAA	
IFb-csy1-F<br>IFb-csy1-R	ACAACTTACCTTTTCCGAGCA<br>AACTCGCCTGTTCCTAACGT	1256
IFb-csy2-F<br>IFb-csy2-R	TCAGGATTATTACGCCAGCCT<br>CGATTTCCCCATGACGTACCA	1244

Table S3. GC-MS identification of Tangerine peel extract

Peak	RT	Compound Name	Area	Area Sum %
1	8.23	α-Terpinene	1283771.2	0.94
2	8.44	p-Cymene	5286912.14	3.88
3	8.56	D-Limonene	32207103.93	23.66
4	9.36	γ-Terpinene	9281307.54	6.82
5	16.06	Thymol	3723304.93	2.74
6	16.40	p-Vinylguaiacol	1234693.53	0.91
7	18.08	Copaene	7117477.17	5.23
8	18.48	Valencene	2147443.79	1.58
9	19.22	Caryophyllene	3808316.3	2.8
10	20.08	Humulene	7936431.09	5.83
11	20.62	γ-Muurolene	1556728.1	1.14
12	20.76	Germacrene D	28005217.4	20.57
13	20.89	β-Selinene	1069517.27	0.79
14	21.07	β-Copaene	1484805.48	1.09
15	21.13	Bicyclogermacrene	2728656.14	2
16	21.19	α-Muurolene	1131878.54	0.83
17	21.54	γ-Cadinene	2983536.5	2.19
18	21.75	β-Cadinene	20160360.62	14.81
19	24.60	5-(1-Piperidinyl)-2-furaldehyde	1512397.17	1.11
20	25.65	Shyobunol	1467472.99	1.08

Table S4. Effect of ZnO/Fe_3O_4 nanocomposites on apoptosis and cell cycle distribution in Caco-2 cells

Condition	viable cells (%)	early apoptosis (%)	late apoptosis (%)	necrosis (%)	G0 (%)	G1 (%)	S (%)	G2/M (%)
untreated	94.7	4.7	0.4	0.1	2.1	34.2	60.6	3.5
Treated	83.6	15.8	0.5	0.0	0.1	1.3	58.1	39.7
